# Structural and Functional Properties of Fiber From Psyllium (*Plantago ovata*) Husk: Current Knowledge and Strategies to Expand Its Application in Food and Beyond

**DOI:** 10.1111/1541-4337.70297

**Published:** 2025-09-23

**Authors:** Lucija Strkalj, Gleb E. Yakubov, Rachel A. Burton, James M. Cowley

**Affiliations:** ^1^ School of Agriculture, Food and Wine The University of Adelaide Urrbrae South Australia Australia; ^2^ Division of Food, Nutrition and Dietetics, School of Biosciences University of Nottingham Loughborough UK; ^3^ Food Biopolymers Laboratory, School of Food Science and Nutrition University of Leeds Leeds UK

**Keywords:** dietary fiber, gastrointestinal health, gluten‐free, hydrocolloids, *Plantago*, psyllium, rheology

## Abstract

Psyllium husk fiber, from the milled surface of *Plantago ovata* seeds, is known for its high viscosity and gelling capacity, making it a widespread health supplement with benefits for satiety, glycemic control, gastrointestinal and cardiovascular health, as well as a texturizer, stabilizer, and fat and gluten replacement. Nevertheless, its application often comes with certain challenges, such as difficulties in adjusting the hydration level, gumminess, and color change. The positive and negative results of the fiber implementation are a consequence of its unique gelation profile and high water uptake. Therefore, a need to uncover the structure and rheological behavior has yielded detailed studies on its fundamental properties, as well as research with the aim of modifying the behavior of fiber for easier application in food products. This review addresses the current literature on the nutritional and health benefits of psyllium, its rheological characterization, and the pros and cons of its application in diverse food groups. Novel approaches to expand its use by modifying its relatively narrow functionality are also suggested, such as fractionation, modification, and genetic strategies. Moreover, a new perspective and future direction for research are discussed, which can also be applied to other highly viscous fibers. This paper serves as an overview of the principal and practical properties of fiber and connects insights from multiple avenues of its exploration with the aim of facilitating its expanded use, not only in food but also in other applications that employ psyllium's unique functionalities.

## 1 Introduction

The *Plantago* genus includes over 200 species, of which *Plantago ovata* is the most researched, understood, and commercially important. *Plantago ovata* is a myxospermous plant, meaning that when its seeds are exposed to water, they produce mucilage—a gel‐like coating rich in polysaccharides that has various hypothesized roles in seed protection and dispersal (Cowley and Burton 2021; Pan et al. 2022). In the case of *P. ovata*, mucilage is derived from the husk—a layer of nearly pure, dried fiber on the seed surface that can be easily removed by stone milling (Phan et al. 2020; Cowley and Burton 2021). The husk, more commonly known as psyllium husk or, simply, psyllium, is widely available and used as a health supplement and food ingredient. Several literature reviews have been published, highlighting the usefulness of psyllium in food products (Belorio and Gómez 2022), describing a broad range of health benefits (Franco et al. 2020; McRorie et al. 2021), and focusing on the bioactive molecules in *Plantago* plants (Zhang et al. 2019). Specific applications in foods include the use of psyllium as a thickener, emulsion stabilizer, and structuring component with a strong capacity to replicate some functions of gluten. For gluten replacement, the key properties of psyllium are the fibrous nature of its gel network (Ren et al. 2020) and its hydrocolloid functionality with high water‐holding capacity. The addition of psyllium markedly improves the acceptability, storage stability, and texture of gluten‐free (GF) bread (Fratelli et al. 2021). Owing to these properties, psyllium is the third most used gluten substitute in the baking industry, after hydroxypropyl methylcellulose (HPMC) and xanthan gum (Belorio and Gómez 2022). In addition, psyllium is used to produce edible and biodegradable food packaging and films; it provides high water vapor permeability and can be combined with other desirable materials, such as proteins (Zhang et al. 2020; Cikrikci Erunsal et al. 2023). Psyllium husk is used for the improvement of gastrointestinal function, and it is well tolerated by people who have irritable bowel syndrome (IBS) (Alhasani et al. 2023; Gunn et al. 2022; Harris et al. 2023; Jalanka et al. 2019; Major et al. 2018; Moayyedi et al. 2019). Due to its satiety effects, psyllium is used for weight loss and management (Gibb et al. 2023). It also has a positive influence on health outcomes on cardiovascular health, including cholesterol levels and glycemic control, and is recommended to people with metabolic syndrome (Pal and Radavelli‐Bagatini 2012).

Despite the high demand for psyllium in an ever‐increasing number of applications, its growing area has not significantly expanded, leading to volatility in the psyllium supply chain. In recent years, export of psyllium by India, the world's largest producer of psyllium, has fluctuated from 32,000 tons to as high as 56,000 tons per year (10‐year standard deviation = 7442 tons) (Govt India Dept. of Commerce 2024). Despite this, the cost per unit (i.e., US$/ton) has increased by 83% over the last decade and is expected to continue to increase. This, coupled with the agronomic constraints and lack of breeding technologies for *P. ovata*, highlights the need to further develop the species as a crop and build technologies, knowledge, and processes that might make psyllium application more efficient (Cowley and Burton 2021; Herliana et al. 2023). In this review, the in‐depth characterization and practical applications of polysaccharides derived from *P. ovata* mucilage are summarized, and strategies to expand their use in food and potentially other product categories are discussed.

Psyllium has been the subject of scientific investigation for decades, and several reviews have addressed aspects of its health benefits or technological applications (Franco et al. 2020). Existing papers often focus on specific health outcomes, such as its role in postprandial glucose response, weight regulation and satiety, or gastrointestinal health (McRorie et al. 2021; Chen et al. 2022; Gibb et al. 2023; Gholami and Paknahad 2024), while others concentrate primarily on its incorporation into food products (Belorio and Gomez 2022). In addition, certain review papers offer insight into the biology and structure of the psyllium plant and fiber (Zhang et al. 2019; Cowley and Burton 2021). In contrast, the present review integrates multiple perspectives, providing a comprehensive synthesis of psyllium's physiological effects and its functional properties in food systems. Furthermore, it includes an in‐depth discussion of psyllium rheology, a topic that, to our knowledge, has not been covered in dedicated reviews, and proposes practical, evidence‐based strategies to address challenges in its application.

Finally, in this review, the aim was to bridge the gap between understanding the intricately layered structure of *P. ovata* mucilage and its functionality, highlight the challenges of its implementation, and to offer solutions to improve certain aspects of its use in food products.^1^

## 2 Nutritional and Health Benefits of Psyllium

**FIGURE 1:**
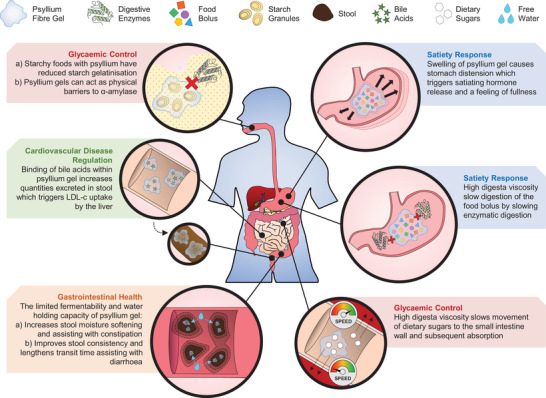
Schematic summary of the dietary fiber‐related health benefits of psyllium consumption.

The first mention of psyllium for medicinal and pharmaceutical purposes was published in 1927 (Nadkarni 1927). Since then, numerous studies have reported the beneficial effects of psyllium on cardiovascular and gastrointestinal health, particularly related to its very high fiber content (>90%). In this review, the focus is on four major health effects of psyllium and how its structural properties determine the underpinning mechanisms. The primary focus of this section is the mechanisms and pathways that are connected to the main health benefits of psyllium (Figure 1). For more detailed information related to hormone regulations and mechanisms linking the primary biophysical and physiological responses with health benefits, the readers can be referred to several published reviews on this topic (Chen et al. 2022; McRorie et al. 2021).

### 2.1 Satiety Control and Weight Management

Psyllium has a long history as an ingredient or supplement to promote satiety and help with weight control. Its primary effects of viscosification and water absorption are important as they result in increased chyme (the mixture of partly digested food and gastric juice) viscosity, which then triggers a number of mechanisms related to satiety (Pal and Radavelli‐Bagatini 2012). For comparison, 2 g of psyllium/100 g of water has significantly higher viscosity (48.40 Pa·s [Pascal·second]) than oat bran, wheat bran, or rice bran (at 1.36, 0.51, and 0.10 Pa·s, respectively). Moreover, during simulated digestion (after 4 h of gastric digestion), the viscosity of a psyllium‐containing solution was 16.15 Pa·s, while the viscosity of oat bran, wheat bran, and rice bran formulations was significantly lower (0.74, 0.64, and 0.54 Pa·s, respectively) (Dikeman et al. 2006; Pavlovich‐Abril et al. 2012). It should be noted that psyllium and the aforementioned brans are different in solubility—the brans are not soluble, while psyllium is considered to be soluble (i.e., it forms a gel). However, these are commonly used fiber supplements, and their comparison shows that psyllium has extremely high viscosity, which is preserved throughout the simulated digestion. High chyme viscosity slows interactions between enzymes and macronutrients; thus, the macronutrients are less hydrolyzed and reach the small intestine in a more intact form. The extended presence of nutrients in the small intestine leads to two physiological outcomes: release of appetite‐regulating hormones and induction of the ileal brake mechanism (Franco et al. 2020; Kristensen and Jensen 2011). Appetite‐regulating hormones, such as cholecystokinin, glucagon‐like peptide 1, and peptide YY, are released into circulation, acting on the nervous system where they promote feelings of satiety, reducing hunger. The ileal brake mechanism is triggered when higher amounts of undigested nutrients reach the distal ileum. This causes a distal‐to‐proximal feedback mechanism that slows the transit of a meal through the gastrointestinal tract to optimize nutrient digestion and absorption (Maljaars et al. 2008). The ileal brake can cause a number of cascade effects, all resulting in the increased small intestinal transit time: reduced jejunal motility, delayed gastric emptying, and reduced secretion of pancreatic enzymes, bile acids, and gastric acid (Maljaars et al. 2008). All these mechanisms contribute to increased satiety and reduced energy intake, which ultimately results in weight control. When compared to guar gum, psyllium ingestion provided sustained weight loss and, compared to a restrictive diet, showed greater weight loss during a 6‐month study (Cicero et al. 2010). More precisely, McRorie et al. (2021) have surmised that daily intake of 6.8–7.6 g of psyllium could significantly reduce the feelings of hunger. A newly published meta‐analysis has found that psyllium taken before meals (mean dose [MD] = 10.8 g/day for 4.8 months) can reduce body weight (MD = −2.1 kg), body mass index (MD = −0.8 kg/m^2^), and waist circumference (MD = −2.2 cm) in overweight and obese participants (Gibb et al. 2023).

### 2.2 Cardiovascular Health

Cardiovascular diseases (CVDs) are the leading cause of death worldwide (Goldsborough et al. 2022); thus, it is vital to understand and apply the preventative role of dietary fiber. It has been shown through clinical trials that psyllium supplementation successfully lowers total and low‐density lipoprotein (LDL) cholesterol (the “bad” cholesterol associated with atherosclerosis), while not affecting high‐density lipoprotein (HDL) cholesterol (the “good” cholesterol that promotes cholesterol excretion) (McRorie et al. 2021). The lowering effect can be explained by two mechanisms: reduced digestion of lipids and increased synthesis of bile acids. Both mechanisms are thought to be related to viscosification. Due to higher digesta viscosity, lipases and lipids will have decreased interactions, resulting in reduced digestion and absorption. Bile acids are produced by the liver and released into the small intestine (duodenum), where they emulsify lipids and enable their digestion. Around 95% of bile acids are recovered in the terminal ileum and reused for the next digestion cycle. When chyme containing psyllium passes through the small intestine, viscous psyllium gels entrap bile acids, leading to their excretion, and therefore, they are not reabsorbed and reused. This causes a reduction in LDL cholesterol level in the blood as the liver uses LDL to produce bile acids, and thus, this positively affects the cardiovascular system (Cai and Chen 2014). A randomized study compared the impact of the wheat dextrin and psyllium supplementation (3.5 and 3.4 g/day, respectively) in hypercholesterolemic adults over 3 months (McRorie et al. 2017). The results showed a 17% LDL reduction and an 11% total cholesterol reduction in a subject group given psyllium, whereas the wheat dextrin group showed no significant change. Another study reported a 7.7% decrease in total cholesterol and 10.7% decrease in LDL cholesterol when 7 g of psyllium/day was given to dyslipidemic children and adolescents (6–19 years) (Ribas et al. 2015). To contextualize how these findings could impact the economy, a cost–benefit analysis concluded that an intake of 7 g psyllium/day in certain patient groups (>45 years, LDL cholesterol level 130 mg/dL) could save, on average, USD 870 million per year in healthcare costs (data for 2013–2020 for the United States) (Shanahan et al. 2019). It is evident from the literature that dietary fiber needs to be highly viscous to elicit a cholesterol‐reducing effect. So far, only two fibers are FDA approved and can have the claim of reducing the risk of CVDs by lowering serum cholesterol level: psyllium and β‐glucan, both potently viscosifying, acid‐resistant soluble fibers (Food and Drug Administration 2002).

### 2.3 Glycemic Control and the Effect on Starch

Elevated blood glucose level is one of the major risk factors for developing metabolic syndrome, CVDs, insulin resistance, and diabetes mellitus type 2 (DMT2). More precisely, postprandial blood glucose level is a better predictor for metabolic syndrome development than the fasting glucose level (Giacco et al. 2016). Therefore, it is important to consider the health implications when ingesting carbohydrates, which are the primary macronutrient driving dose‐response reactions for blood glucose (Giacco et al. 2016). Implementing psyllium into a diet can help lower both fasting and postprandial blood glucose levels, with the most prominent effect on people with DMT2 or with developing DMT2 (McRorie et al. 2021). As with other health benefits where psyllium's viscosification effect reduces nutrient digestion/absorption, chyme containing psyllium shows slower glucose diffusion and absorption, which decreases the postprandial peak. Several reviews have been published on the topic of psyllium's influence on glycemic control. Mostly, it has been concluded that psyllium has a positive (decreasing) effect on blood glucose levels, but its impact on insulin levels is less conclusive. A review published by Pal and Radavelli‐Bagatini (2012) has highlighted that psyllium intake of 10–14 g/day for 6–8 weeks reduced glucose levels, but increased psyllium intake (21–23 g/day, for at least 12 weeks) reduced plasma insulin levels. However, longer studies (6 months) have shown that the lower doses of psyllium (10 g/day) can also decrease both glucose and insulin levels (Cicero et al. 2010). Gibb et al. (2015) concluded that psyllium supplementation can decrease fasting glucose, levels of HbA1c (glycosylated hemoglobin widely used as a biomarker for DMT2), and postprandial glucose in diabetic patients, and psyllium also has a reducing effect on insulin and postprandial glucose in healthy individuals. Lastly, a more recent meta‐analysis revealed that psyllium dosage of more and less than 10 g/day lowered fasting blood glucose, HbA1c, and Homeostatic Model Assessment of Insulin Resistance (quantitative assessment of insulin resistance and pancreatic beta‐cells function) but not if the intervention was shorter than 50 days (Gholami et al. 2024). Moreover, the same study concluded that insulin levels were not significantly reduced by psyllium consumption. Overall, psyllium shows a positive effect on blood glucose homeostasis.

Besides its physiological effect, psyllium affects starch behavior in food processing, which should be applied when developing or improving food formulations (Z. Zhou, Ye, et al. 2022; Cowley et al. 2025). Starch is a complex glucose‐containing carbohydrate and a dietary staple all over the world, constituting the most important source of energy for humans. Its digestibility and glucose release depend on many factors (such as amylose to amylopectin ratio, type of processing, gelatinization and retrogradation, etc.), but this discussion shows how soluble viscous fibers, such as psyllium, can alter the susceptibility of starch to digestive amylases (Singh et al. 2010; H. Zhang et al. 2022). Briefly, the starch granules become more accessible to amylase when gelatinized (i.e., hydrothermally treated) because the ordered crystalline structure of starch granules is degraded. As with other hydrocolloids, combining psyllium with starch could potentially (1) limit their gelatinization extent by physically restricting granule swelling and/or (2) create a network around the starch granules that acts as a mechanical barrier preventing amylase from reaching the granule (H. Zhang et al. 2022). This was explored in wheat, potato, and tapioca starches, and it was shown that psyllium led to a decreased rate of starch digestion by restricting granule swelling and gelatinization and maintaining the ordered structure of starch granules (Sevilmis and Sensoy 2022). When comparing psyllium and cellulose fibers (both at 10%) added to bread and crackers, results showed that psyllium reduced levels of rapidly digested starch and increased slowly digested starch in both products, while cellulose fibers had no significant effect (Bilgic and Sensoy 2023). Even though these studies employed in vitro digestion models, they highlight the importance of viscous fibers in controlling glycemic index (GI) of foods and constitute a factor that should be considered in food processing.

### 2.4 Gastrointestinal Health

It is recommended that the daily intake of fiber for adults should be 30 g or more (per 2000 kcal/day) (McKeown et al. 2022). However, the type of fiber is important when distinguishing its nutritional benefits, as structural properties dictate its functionality and mechanisms through which fiber achieves certain health effects (Meldrum and Yakubov 2025). Fibers are usually categorized as soluble and insoluble; however, that does not reflect on their health functionality (Gidley and Yakubov 2019). Soluble fiber is chemically characterized as soluble or gel‐forming in water, but it can be viscous or nonviscous. Fibers that are viscous provide cholesterol‐lowering and satiety effects (McRorie and McKeown 2017). Insoluble fiber is not water soluble and is not viscous; therefore, it does not show health benefits that are related to viscosity (McRorie and McKeown 2017). Fiber can be fermentable or nonfermentable, and the topic of psyllium fermentability has been discussed. Psyllium is a soluble and highly viscous fiber with a range of benefits and applications targeted toward the health of the gastrointestinal tract (Gibb et al. 2023). Psyllium administration is therapeutic for both constipation and diarrhea. Psyllium gel has a high water‐holding capacity, which withstands dehydration in the large intestine, increasing stool weight and acting as a softener and emollient (McRorie and McKeown 2017). An MRI study in humans showed that psyllium intake (7 g, three times a day) increased the water content in the small bowel, colonic volume, and T1 measurement (determination of longitudinal relaxation time which describes the recovery of magnetization along the direction of main magnetic field), which correlates with free water measurement and water content of the stool (Major et al. 2018). In a healthy (non‐constipation/diarrhea) population, 1 g of psyllium increases stool weight by 5.9–6.1 g, which is higher than wheat or oat bran (4.9–5.4 and 3.4–4.5 g, respectively) (Wärnberg et al. 2009). It can be concluded that the laxative effect of soluble fiber (such as psyllium) is a result of increased stool moisture. Conversely, the laxative effect of insoluble fiber (such as cereal brans) is caused by its mechanical irritation effect on the bowel mucosa. Mucous membranes then release water, as a defense reaction against the irritation, which increases the water content of the stool (McRorie and McKeown 2017). For patients suffering from diarrhea, psyllium also offers relief, as the high water absorption capacity (WAC) and increased colon transit time allow for appropriate stool formation (Wärnberg et al. 2009).

Considering its beneficial effect on constipation and diarrhea, and the gentle nature of its effects (i.e., nonabrasive effect on mucous gut membrane), it is justified that psyllium is a recommended therapy for patients with IBS, who can experience periods of both constipation and diarrhea. Per the American College of Gastroenterology, psyllium is the only recommended fiber for IBS and constipation treatment (Ford et al. 2018). It is also recommended by the Japanese Society of Gastroenterology and Canadian Association of Gastroenterology as an IBS treatment (Fukudo et al. 2021; Moayyedi et al. 2019).

Nevertheless, fermentability of psyllium husk and its polysaccharides has been a topic of debate. As mentioned, psyllium is considered to be a soluble fiber, and other soluble fibers are associated with fermentability, such as pectin or guar gum, though the soluble/insoluble definitions are complex (Gidley and Yakubov 2019). Indeed, in vitro data have indicated psyllium has some level of fermentability (Campbell and Fahey 1997), and in vivo studies in constipated patients have shown production of short‐chain fatty acids (SCFAs) and changes in bacterial populations when psyllium was added to a diet (Jalanka et al. 2019). However, most research suggests that psyllium is mostly nonfermentable or slowly fermentable due to three reasons: its gel structure prevents bacteria from entering the substrate; substitution density limits backbone access by enzymes; and densely branched structure presents steric hindrance to human intestinal and microbiota enzymes (Marlett and Fischer 2003; McRorie et al. 2021). Marteau et al. (1994) found that psyllium appeared still intact in stools, which contributed to its bulking effect, but it also led to higher amounts of acetate, propionate, and total SCFAs, implying that fermentation was elevated. Psyllium consumption (15 g/day, for 12 days) is also shown to increase apparent viscosity of aqueous stool extract and produce bulkier and more moist stools when compared to other fiber sources in corresponding amounts (Marlett et al. 2000). Researchers were able to extract fibrous gelatinous mass from the stool of participants who consumed psyllium, while the control group did not have the gelatinous fraction of the stool. It was concluded that a fraction of psyllium is not fermented (Marlett et al. 2000). In a randomized controlled trial studying fecal incontinence, it was reported that only psyllium (16 g of fiber per day for 32 days, compared with placebo, carboxymethylcellulose, or gum arabic) resulted in a visible gel in a stool (Bliss et al. 2014). The formation of a gel in feces and the “firming consistency” of psyllium was proposed as a mechanism for a decrease in fecal incontinence frequency. It was explained that the amount of psyllium is an important parameter—16 g psyllium per day provided benefits that were not seen when participants took 7.1 g psyllium per day (Bliss et al. 2001). It was concluded that both the SCFA production and the unfermented fiber presence contribute to the improvement of fecal incontinence (Bliss et al. 2001; Bliss et al. 2014). In their review, McRorie et al. (2021) have discussed the fermentability of psyllium and concluded that it is not fermentable and does not cause excess gas in in vivo studies. Moreover, it should be highlighted that in vitro studies are mostly done at low concentrations to prevent gel formation and to allow for constant mixing, which could lead to fermentability but does not reflect in vivo conditions when psyllium is consumed (McRorie et al. 2021; Slavin and Green 2007). Interestingly, a recent study (Alhasani et al. 2025) showed that psyllium slowed down the colonic transition of inulin but did not change fermentation over a 24‐h period. It seems that fermentability could be influenced by the solubility of psyllium fiber, which will be discussed later in Section 5.2. Overall, perhaps the fermentability of psyllium would be best described as partially fermentable, as it was shown that the amount (dose of psyllium per day), preparation (on its own or with other fibers), and extraction (depending on the temperature) affect its fermentability.

An important aspect to mention is psyllium's modulation of the gut microbiome, supporting a healthy gut and function. Recent clinical trials demonstrate that psyllium supplementation not only alleviates constipation by increasing stool water content but also beneficially modulates the gut microbiota, promoting butyrate‐producing and SCFA‐associated taxa (e.g., *Faecalibacterium*, *Lachnospira*, *Roseburia*) while reducing bacteria linked to hard stools and slow transit (*Christensenella*). These microbiota shifts correlate with improvements in stool frequency and consistency, with effects more pronounced in constipated individuals than in healthy controls, supporting a role for psyllium in long‐term gut health. A 2019 randomized controlled trial found that 7 days of psyllium in constipated patients increased stool water, enriched SCFA‐producing bacteria (*Phascolarctobacterium*, *Veillonella*, and *Sutterella*), reduced taxa linked to hard stools (*Christensenella* and *Coriobacteria*), and improved gut transit (Jalanka et al. 2019). Next, a clinical study in women with chronic constipation found that psyllium improved symptoms by increasing stool water and bulk while shifting the gut microbiota toward populations linked to better bowel function, such as *Faecalibacterium* (Yang et al. 2021).

Lastly, an important aspect of psyllium use in gastrointestinal health is its application as an encapsulating agent. Due to its strong gelling nature and resistance to acidic conditions of the stomach, it can be used for the delivery of probiotics and prebiotics (Martellet et al. 2022). Encapsulation possibilities of psyllium could help in the treatment of gastrointestinal diseases, contribute to microbiome variability, and be useful in personalized medicine development.

## 3 *Plantago* Mucilage Extraction and Subsequent Chemical and Rheological Characterization

### 3.1 *Plantago* Mucilage Structural Properties

**FIGURE 2:**
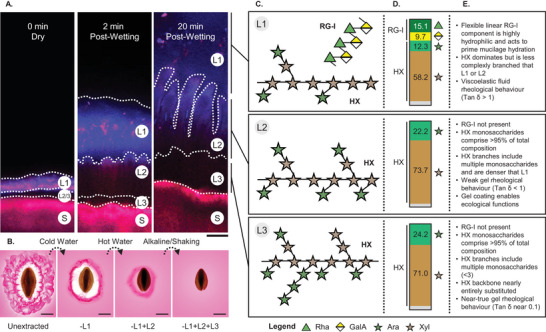
Combining microscopy, chemical, and rheological analyses has enhanced the understanding of *Plantago ovata* mucilage release and physicochemical properties. (A) When dry, the laminated mucilage polysaccharides in the layers are thin and dry. When this layer is milled dry, it comprises psyllium husk (Layer 1 [L1] + L2/3 with some insoluble residue). Upon wetting, the different layers hydrate and expand away from the seed (S) depending on chemistry and solubility. Scale = 100 µm. Adapted from Phan et al. (2020). (B) Three layers (L) appear to be removed with sequential extraction in *Plantago ovata*. Acidic polysaccharides stained with ruthenium red. Scale = 1 mm. Adapted from Cowley et al. (2020). (C) Schematic representations of rhamnogalacturonan‐I (RG‐I) and heteroxylan (HX) polymers present in three sequentially extracted fractions of *Plantago ovata* mucilage based on monosaccharide and linkage data from Yu et al. (2017). Monosaccharide locations do not correspond with true linkage configuration and are demonstrative only. (D) Share of monosaccharides in each fraction in molar ratios. Non‐RG‐I and HX monosaccharides were combined in gray. Data adapted from Yu et al. (2017). (E) Synthesis of information on composition, structure, rheology, and function from multiple studies including Yu et al. (2017). Note that layers may not be as discrete as the three‐layer model presented here and that a wider range of extraction conditions has yielded additional fractions that follow the same chemical and rheological trends discussed here (Ren et al.^2020^). It is possible that these core layers can be sub‐fractionated. Psyllium husk is therefore a heterogenous mixture of the same polymer types of different complexity, with some insoluble residue.

The extractability of fibers is largely determined by their chemical structure and properties. Most of the scientific literature regarding *Plantago* mucilage reports the use of varying solvents for polysaccharide extraction, while works using ultrasound, microwave, high pressure, and similar methods are sparse. The type of solvent affects the composition of the extract and, consequently, its characteristics (Zhang et al. 2019). For example, if cold water (CW) is used for mixing the husk or the seeds, only the fiber fraction that is soluble under those conditions will be solubilized, while the rest will retain its gel formation. In psyllium, CW extraction has been shown to yield fiber with slightly negative charge and low gelling capacity (Yu et al. 2017; Zhou et al. 2020). On the other hand, the extraction with alkaline solvent will result in a higher fiber yield, as increased pH can disrupt the chemical bonds and network formation, swelling, and charge (Tejada‐Ortigoza et al. 2016). In psyllium, alkaline‐extracted fiber showed high gelling capacity and strong viscoelasticity (Yu et al. 2017). In this section, an overview of *Plantago* mucilage extraction is presented, and a corresponding polysaccharide structure is discussed. These details are combined graphically in Figure 2, along with an overview of their rheological properties, which are discussed in Section 3.2.

The first *Plantago* species whose mucilage composition was researched was *Plantago psyllium* (Anderson and Fireman 1935), though subsequent papers refer to the species studied as *P. ovata*. This might reflect the common confusion between blond psyllium (*P. ovata*), the source of psyllium husk, and a much less common relative, dark psyllium (*P. psyllium* syn. *P. afra*), which does not possess a husk removable by milling. Irrespective of this, it was reported that the mucilage consists of D‐xylose, l‐arabinose, and d‐galacturonic acid, with its exact composition being dependent on the extraction conditions (temperature, seeds:H_2_O ratio, time, and force applied to push the gel through the cloth) (Anderson and Fireman 1935). Composition of aqueously extracted mucilage from *Plantago lanceolata* (Mullan and Percival 1940) and *Plantago arenaria* (Hirst 1951) was described as “similar,” containing xylose, arabinose, and galacturonic acid, along with d‐galactose, which had not been reported before. The first reported multistep extraction of *Plantago* mucilage consisted of extracting *P. ovata* husk in H_2_O at 15°C and then again at 90–95°C. The CW extract had a higher uronic acid content and lower pentosan content than the hot water (HW) extract, and the authors concluded that mucilage most likely consists of two distinct polysaccharides—one that is readily soluble in water and rich in uronic acids, and the other that is neutral (Laidlaw and Percival 1949). Detailed structural analyses were required to determine how the constituent monosaccharides detected in these studies were assembled.

Kennedy and others (Kennedy et al. 1979; Sandhu et al. 1981) studied the structure of alkaline‐extracted *P. ovata* mucilage (1.2 M NaOH) using methylation analysis. They showed that the polysaccharide is highly branched (32.4% of all residues were branching points), but their proposed structure of a single polymer was later proven to be incorrect (xylan backbone with (1→3) and (1→4) linkages substituted at *O*‐2 and *O*‐3 with arabinose, xylose, and galactopyranosyluronic acid‐rhamnose). They were also the first to suggest that *P. ovata* gel most likely does not have covalent bonds. This was proven much later by Yu et al. (2017) and will be discussed later in detail.

One of the early papers that described mucilage characteristics for multiple *Plantago* species utilized one‐step acidic extraction with HCl (0.1 N) for 11 different types of *Plantago* seeds (Sharma and Koul 1986). Importantly, the authors discovered that mucilage mass does not correspond to its water swelling properties—*P. ovata* had the highest mass of extracted mucilage, while *P. psyllium* and *Plantago indica* had a higher swelling factor (17.20 and 15.70, respectively), followed by *P. ovata*, whose swelling factor was 15.25 mL/g. This gave an early indication that even though *P. ovata* is the most widely used *Plantago* species, other cultivars have potential for food and nutritional purposes.

Based on monosaccharide composition and glycosidic linkage data from husk extracted with hot water (IHWE) and alkali (IHAE), Edwards et al. (2003) proposed an updated structure for arabinoxylans (AXs) of *P. ovata* husk: the backbone is formed of β‐d‐(1→4)‐linked xylopyranoses to which α‐l‐arabinofuranoses are attached via α‐(1→3) and α‐(1→2) linkages. The AX is highly branched with backbone substitutions (at a density that depended on extraction method), including α‐d‐galactopyranuronic acid (GalA*p*)‐(1→2)‐linked‐α‐l‐rhamnopyranose‐(1→4)‐β‐d‐Xyl*p*, α‐d‐GalA*p*‐(1→3)‐linked‐α‐l‐Ara*f*‐(1→4)‐β‐d‐Xyl*p*, and α‐l‐Ara*f*‐(1→3)‐linked‐β‐d‐Xyl*p*‐(1→4)‐β‐d‐Xyl*p*. This was also the first study to report the structural differences and molecular weights of fractionated psyllium husk polysaccharides. IHWE had a molecular weight of 2200 kDa, with only 18% of its xylose backbone residues unsubstituted, 31% monosubstituted, and 11% disubstituted, whereas IWAE has a molecular weight of 1600 kDa, with 20% of the xylose units unsubstituted, 31% monosubstituted, and 18% disubstituted. It has since been found that the α‐d‐galactopyranuronic acid (GalAp)‐(1→2)‐linked‐α‐l‐rhamnopyranose identified is derived from a linear rhamnogalacturonan‐I type pectin polymer that is highly hydrophilic and acts to initiate hydration of the main structural xylan‐type polymers (Phan et al. 2020; Yu et al. 2017; An et al. 2026).

A similar structure for alkali‐extracted *P. ovata* mucilage was published by Fischer et al. (2004): β‐(1→4)‐linked d‐xylopyranosyl units forming the backbone, and single xylopyranosyl side chain residues at *O*‐2, with trisaccharide l‐arabinofuranosyl‐(1→3)‐d‐xylopyranosyl‐(1→3)‐l‐arabinofuranosyl side chains at position *O*‐3. The authors developed a multistep alkaline extraction process for *P. ovata* mucilage and reported the fractions to be neutral. Based on previously published results, some have reported the mucilage to be acidic, while others found it to be neutral. It is sensible to assume that this discrepancy is a consequence of different extraction methods, and this was later confirmed by Guo et al. (2008). They reported structural data for three mucilage fractions (soluble in H_2_O at 80°C [AE], soluble in 0.5 M NaOH [AES], and a gel fraction in 0.5 M NaOH [AEG]). AE and AES had up to 15% uronic acids, but AEG was neutral. The solubility and gelling properties were suggested to be explained by differences in side chain motifs of the three fractions—branching of the three fractions was 60%, 32%, and 74% for AE, AES, and AEG, respectively. AE had the most complicated and longest side chains (up to three monosaccharide subunits), and AEG had short and regular side chains, which could allow closer intermolecular interactions and explain its high gelling capability.

It is important to note that the xylans in *P. ovata* mucilage are different from cereal bran xylans, which are considered true AXs (Kiszonas et al. 2013), because, although their chemistry is similar, it is distinct. While both contain xylose backbones, cereal bran xylans are substituted solely with arabinose residues, making them true AXs. On the other hand, *P. ovata* mucilage xylan side chains are much longer and contain xylose. This is considered to preclude the *P. ovata* polysaccharide from being defined as a true AX, as it is often labeled, and it should more correctly be classified as heteroxylan (HX). Second, most backbone residues of *P. ovata* HX are substituted, while unsubstituted xyloses are prevalent in cereal bran AX. Lastly, *P. ovata* mucilage HX shows decreasing water solubility with increasing arabinose to xylose (A:X) ratios, which is opposite to the behavior of cereal bran AX. These findings point to a set of structurally diverse HX molecules comprising *P. ovata* mucilage, likely applicable to the HX of other *Plantago* species, that confer highly unique functionalities.

### 3.2 *Plantago* Mucilage Functional Properties

As newfound knowledge of the chemistry of whole and fractionated *P. ovata* mucilage polysaccharides was acquired, the interest in their secondary structure and intermolecular interactions grew. More detailed information could unlock the drivers behind its unique gelling behavior and guide its use in providing beneficial health properties and applicability in food, pharmaceutical, and engineering industries. Therefore, the attention was rapidly focused on the rheological properties of *P. ovata* mucilage.

In one of the earliest studies of its rheology, *P. ovata* mucilage was compared to the xanthan gum (Haque et al. 1993). Alkali‐extracted (2.5 M NaOH, room temperature) *P. ovata* husk mucilage was described as a weak gel whose structure is broken down under steady shear rotation but is retained under small deformation oscillatory measurements. In addition, melting of the gel was described as a continuous process happening above 80°C.

Since Haque et al.’s pioneering study, Farahnaky et al. (2010) have employed amplitude (strain) sweep, frequency sweep, and temperature sweep tests under varying conditions (temperature and pH) to assess the rheology of *P. ovata* mucilage, which was presumably extracted at 25°C, although the method section is lacking in detail. As expected, an increase in the gel concentration from 2% to 2.5% and 3% resulted in moduli increase due to higher viscosity and the interactions and entanglements between the polymer chains. *G*ʹ, which is the measure of deformation energy stored in the sample during the shear measurement, that is, the elastic behavior of a sample, was higher than *G*ʺ—the measure of deformation energy lost in the sample during the shear process, that is, the viscous behavior of a sample, at all tested concentrations. Frequency sweep tests showed *G*ʹ and *G*ʺ curves almost parallel to each other, thus displaying characteristics of a weak gel, as opposed to entanglement systems, which display an intersection of *G*ʹ and *G*ʺ. Moreover, tan *δ* (the ratio between viscous and elastic behavior, i.e., *G*ʺ:*G*ʹ) for all concentrations was lower than 0.2, indicating a weak gel structure, since tan *δ* >1 reflects a more liquid‐like material, while tan *δ* <1 reflects a more solid‐like material. Temperature sweeps showed a long and continuous melting process where *G*ʹ and *G*ʺ of the 2.5% gel showed a plateau at temperatures above 40°C. Frequency sweep tests of a 2.5% gel at different pH values showed decreased *G*ʹ and *G*ʺ but higher tan *δ* at low pH (2.5), indicating that the gel has lost its elasticity due to lower electrostatic repulsion at lower pH values.

A series of papers published by Yu et al. (2018, 2017, 2019) presented in‐depth structural and rheological characterization of *P. ovata* mucilage, as well as proposed mechanisms that govern the unique behavior of different mucilage fractions. Three fractions were isolated, namely a CW fraction extracted with H_2_O at 25°C, an HW fraction extracted with H_2_O at 65°C, and a KOH fraction extracted under alkaline conditions with 0.2 M KOH. Their monosaccharide composition agreed with previously published results—the CW fraction had the highest amount of acidic sugars and rhamnose, while the A:X ratio increased along with increasingly intense extraction steps, giving A:X ratios of 0.20, 0.30, and 0.33 for the three consecutive fractions. HW and KOH fractions had strikingly similar monosaccharide composition, linkage analysis patterns, and molecular weight (HW—971 kDa, KOH—953 kDa); however, their rheological characteristics were very different. The CW fraction can be described as a viscoelastic fluid (tan *δ* > 1). Although HW and KOH fractions both display gel‐like behavior, KOH is frequency independent (thus can be classified as a true gel), whereas HW is frequency dependent (meaning that it is more liquid‐like than KOH). Moreover, KOH gels retain their mechanical properties up to 85°C, but melting of the HW fraction begins at 41°C ± 2°C, and HW shows a gradual decrease in *G*ʹ as the temperature increases. The authors have proposed that both gels are physical, meaning that hydrogen bonding is responsible for the gel‐like properties, as opposed to covalent bonding. To show this, experiments using 8 M GuHCl (a strong chaotropic agent that disrupts hydrogen bonding) proved to have significant effects on the rheological profile, and it dissolved gels from both fractions. Since ferulic acid residues are a primary driver for covalent bonding in AX gels (as shown for cereal AXs) (Kiszonas et al. 2013), and *P. ovata* HX contains very low amounts of ferulic acid residues (Yu et al. 2017), the proposed reason for the different behaviors of HW and KOH fractions is attributed to differences in their side chain distribution and hydrogen bonding of side chain to another side chain or to the backbone, which then drives its rheological behavior (Yu et al. 2018). Moreover, it was shown that differences in temperature or solvents used have an impact on the intricate structure and polysaccharide motifs, and removal of terminal arabinoses in side chains dramatically influences rheological behavior, thus confirming that hydrogen bonding of side chains is the main force driving the gelling behavior of these mucilage fractions (L. Yu et al. 2019).

In a study by Ren et al. (2020), five different fractions of *P. ovata* husk mucilage were aqueously extracted under increasing temperatures (20°C, 40°C, 60°C, 80°C, and 100°C). It was reported that with temperature increase, A:X ratios increased as well (0.298, 0.305, 0.322, 0.363, and 1.979, respectively), indicating very different polymers that have correspondingly different functional properties. F20 (a fraction extracted at 20°C) was the only fraction that was influenced by thermal treatment—its mechanical spectrum showed differences before and after heating, while mechanical spectra of other fractions overlapped. The authors have proposed that F20 might be substituted with single xylose units and simple side chains, which can partially associate and form a weak gel at low temperature, but are influenced by higher temperature and, therefore, show differences before and after first and second heating. On the other hand, fractions extracted at higher temperatures had more complicated side chain motifs that restrict associations at higher temperatures and are probably supported by hydrogen bonds, thus forming a “physical” gel that retains its rheological properties even after first and second heating.

Similarly, P. Zhou et al. (2020) have characterized two fractions from *P. ovata* husk mucilage. A cold‐extracted fraction (CP, 25°C) had five times more uronic acids than a hot‐extracted fraction (HP, 85°C) at 18.5% and 3.5%, respectively. The molecular weight of CP was 630 kDa, while that of HP was 2100 kDa. CP exhibited behavior consistent with a viscoelastic fluid, while HP displayed weak gel characteristics and had a critical gelation point of 4.8 mg/mL. Temperature sweep tests showed thermoreversible properties of both fractions, but melting temperature and gelation temperature showed a dependence on concentration. This supports the previously proposed theory of Yu et al. (2018) that weak gel behavior is dictated by hydrogen bonds and polymer entanglements. Thus, it is possible that below gelation temperature, the gel‐like behavior of HP was facilitated by hydrogen bonds, but above gelation temperature, the polymers exhibit more movement, which contributes to viscous behavior. Furthermore, steady flow sweep of CP showed shear‐thinning behavior and increased apparent viscosity with an increase in concentration, which can be explained by more random chain entanglements at higher concentrations that are then susceptible to shear.

With evidence that hydrogen bonds play a key role in the gelling behavior of *P. ovata* mucilage, it was then shown that these bonds can be described as “weak” and “strong” in AX‐W (AX extracted with H_2_O at 65°C after extraction at 25°C) and in AX‐A (AX extracted with KOH), respectively (Yu et al. 2021). By utilizing a wide range of oscillatory measurements, it was possible to measure and calculate the molar energy of hydrogen bond junctions for the hot‐water‐extracted (HWF) fraction (97–144 kJ/mol) and the alkaline‐extracted fraction (AEF) (402–480 kJ/mol). The authors called the weak hydrogen bonds, which can be used to describe the rheological behavior of HWF, as “fast self‐healing,” while strong hydrogen bonds are “slow self‐healing” and characteristic of AEF. Practically, this means that gelling behavior is indeed driven by *P. ovata* polysaccharide motifs, as hydrogen bonds are primarily created between terminal xyloses and arabinoses in side chains, and these differences could and should be exploited in fabricating different food products or packaging materials and for medicinal and pharmaceutical purposes.

In the published literature, multistep aqueous and alkaline extractions of *P. ovata* are the most common, while commercially simpler hydration processes are used. Thus, a need for adjusting the extraction process is emerging. Kaczmarczyk et al. (2023) hydrated husk in H_2_O at 25°C before centrifuging to produce a mucilage‐rich supernatant they refer to as “raw extracts.” For research purposes, reconstituted extracts are used, meaning mucilage is extracted, dried, and then rehydrated to get the exact concentration for further experiments. In this case, the polysaccharide concentration in raw extracts was not presented, so the polysaccharide concentration was not controlled. As concentration is a factor critically influencing rheological properties, the results cannot be interpreted as 1‐for‐1 comparisons of functionality between fractionated polysaccharides but might be insightful to end‐users who wish to perform a simple fractionation to get targeted properties. As such, the study of raw extracts corresponds to industry or household use of psyllium husk. To this end, the authors show that the raw extracts can be described as viscoelastic fluids with two types of polymer chains whose molecular weights are 200 and 1780 kDa.

Mucilage properties can be affected by different conditions before and after extraction, and even before seed harvest. Increased rainfall at *P. ovata* crop maturity leads to excessive moisture at the hydrophilic seed surface, resulting in changes in seed and husk color and a diminished WAC, which is a benchmark of psyllium utility (Cowley et al. 2022). Crops that were subjected to unseasonal rain had lost part of the most soluble outer layers of mucilage, along with increased microbial infestation and poorer germination. Even though the rheological properties of mucilage were not tested, its quantity and quality were impacted; thus, it is reasonable to assume that such exposure would affect the functional properties as well.

As it became clear that extraction methods affect gel properties, tailored methods for research, food, and health applications began to emerge. Cowley et al. (2020) streamlined a small‐scale fractionation process in which three different fractions of mucilage are extracted by shaking the seeds in H_2_O at 25°C, then at 65°C, and finally, seeds were subjected to intense agitation using a tissue disruptor‐type mixer mill. The entire process takes less than 5 h and offers an alternative to alkaline extraction, which can be tedious to neutralize and dialyze. The protocol is applicable not only to *Plantago* seeds but also to other mucilage‐producing seeds when screening myxospermous germplasm to assess seed quality and for chromatographic analyses.

Overall, various extraction processes and fiber characterization have been reported for psyllium. Due to its viscous nature, extracting the gel in its entirety required a multistep extraction. Consequently, fiber fractions were reported to be different in structure and in rheological profile. As shown, there are many research papers describing the structure of said fractions. However, the application of these fractions is still underexplored. It is sensible to assume that fiber fractions with different rheology properties would have different structuring potential in food, which should be further explored. Therefore, the next chapter brings an overview of challenges when applying psyllium in food products, and, further on, solutions are presented based on the variability of extracted psyllium fractions.

## 4 Positive and Negative Impacts of Psyllium Fiber Implementation

Research has shown that most people do not meet the recommended dietary fiber intake: for example, Europeans eat around 78% and Americans 62% of the recommended intake (McKeown et al. 2022). Fiber can be ingested in two ways: in an isolated form as a supplement or in a food product, either added or naturally present. Oat bran and psyllium husk are common examples of fiber supplements, which people usually take with water (as recommended). This is the most common way to assess the health effects of a fiber in clinical studies; however, this is most likely not representative of typical diets consumed by people, unless they have been advised by a health practitioner.

It should be highlighted that most clinical studies are focused on psyllium husk or powder alone, rather than a food containing psyllium. Moreover, long‐term clinical studies that include food products with psyllium are sparse. An early clinical study from 1998 has reported improved serum lipid profile when patients were consuming common food products that were enriched with psyllium (five different ready‐to‐eat cereals, pasta, snack bars, and bread) for 24 weeks (Davidson et al. 1998). Patients consumed 10.2 g of psyllium per day, and their LDL cholesterol was lower by 5.3% when compared to the control group. Another early study included psyllium‐enriched cereals, which were proven to be beneficial in children with hypercholesterolemia (Davidson et al. 1996). After 6 weeks, a 7% reduction in LDL cholesterol was found. However, newer long‐term studies including food products containing psyllium are lacking, and this area of research can be considered underexplored.

**FIGURE 3:**
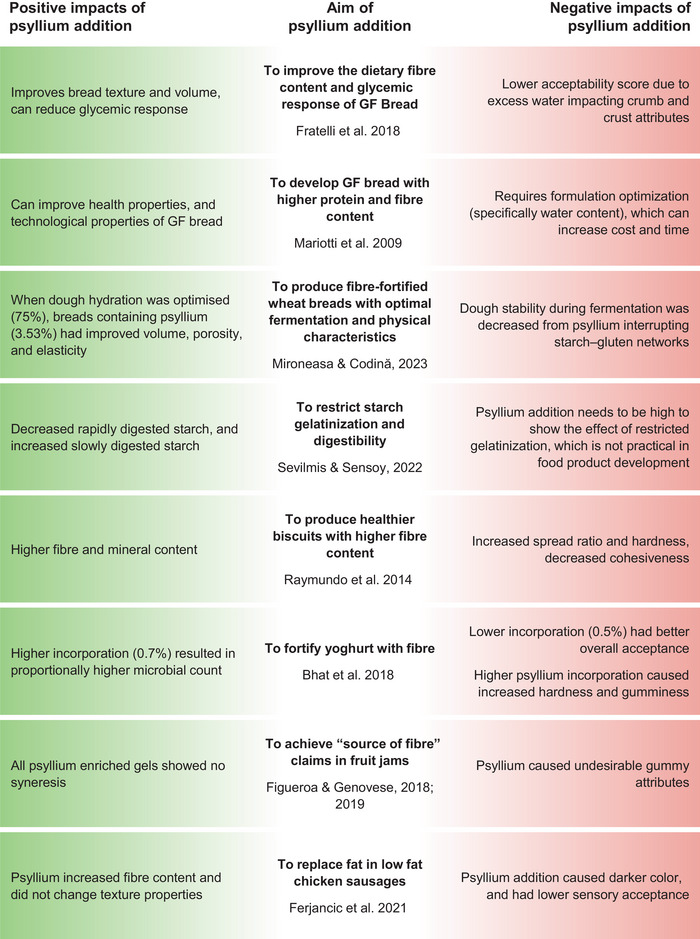
Specific aims of psyllium addition to food products and subsequent positive and negative effect.

Eating whole foods rich in fiber is typically the first advice when recommending increasing fiber intake (McKeown et al. 2022), which is why the food industry frequently aims to enrich food products. Additionally, it has been shown that health claims have an impact on consumers’ choices (Benson et al. 2019). According to EFSA, a food product can have specifically worded claims related to reduction of glycemic response and healthy gastrointestinal function if it supplies 3.5–14 g of psyllium, inulin, oat bran, or flax groats in its daily serving (EFSA 2010). If a food product contains 1–3 g of psyllium seed husk per its daily serving, it can have health claims related to satiety (EFSA 2010). However, adding fiber to a food product can cause negative organoleptic changes (Foschia et al. 2013). Using baked goods as a common fiber‐fortified example, insoluble fiber can cause lower bread volume or darker crust color, while soluble fiber can change dough viscosity and ultimately bread texture. As detailed in the introduction, *Plantago* mucilage has many applications in the food and health industries. Previously described mechanisms (Section 2) behind its unique gelling behavior (Figure 2) are related to its medicinal benefits (illustrated in Figure 1) but also make it difficult to include adequate amounts of fiber in the diet and food products. In this section, the challenges of *P. ovata* husk and mucilage implementation in food are highlighted, followed by the examples of how its unique properties can be both advantageous and adverse (Figure 3).

Psyllium is an important structuring agent in GF breadmaking, but its incorporation requires increased water addition to maintain quality. However, too much water can lead to bake loss, decreased volume, and lower bread acceptability. Fratelli et al. (2018) modeled the effect of addition of various amounts of psyllium (2.86%–17.14%) and water (82.14%–117.86%) in GF bread on organoleptic qualities. The authors showed that up to 10% psyllium can be added without adjusting water content, resulting in acceptable bread with enhanced texture. Nevertheless, bread with 2.86% psyllium and 82.14% water addition had the highest overall acceptability (8.72 on a 10‐point scale). Bread with 17.14% psyllium and 117.86% water had decreased GI in vivo (−25%), but lower acceptability with a score of 7.11. This study highlights the gap between psyllium levels for higher acceptability and those required to improve glycemic response. When psyllium and HPMC are added to GF rice bread, it was found that psyllium decreased bread volume and increased hardness, proportional to its addition (0–4 g/100 g), but higher water addition (90–110 g per 100 g of flour) mitigated the negative traits to a certain extent (Mancebo et al. 2015). The usefulness of psyllium addition in nonmodel GF doughs (corn starch, amaranth flour, pea isolate) was confirmed by Mariotti et al. (2009): 2% psyllium provided a cohesive network to support the starchy matrix and created a rheologically stable system with a longer linear viscoelastic region. It is important to mention that the authors varied water addition, as per farinograph measurements, to ensure a workable GF dough, and thus water deficit was at least partly accounted for. The difference between the quantity of psyllium that would provide desirable products and the amount that would have significant health benefits was also pointed out in a review by Belorio and Gómez (2022). Even so, psyllium continues to be important in the GF bakery industry, but Roman et al. (2019) have highlighted the gap between commercial use and research of psyllium fiber in GF bread. They noted that incorporation of pectin is widely studied, even when it is not found in commercial GF breads, whereas psyllium should be researched more, considering that it is commercially widespread.

Psyllium has also been applied to gluten‐containing products. Unlike in GF breads, where psyllium is a structuring agent, psyllium is added to gluten‐containing products typically to improve nutrition. However, in these systems, there is often competition for water between psyllium and gluten, and optimization is needed. To determine the optimal amount of psyllium addition and hydration in wheat bread, Mironeasa and Codină (2023) have used response surface methodology to model how four levels of psyllium addition (0%, 2%, 4%, 6%) and hydration (60%, 65%, 70%, 75%) impact bread physical properties (retention coefficient, loaf volume, porosity, and elasticity). It was concluded that bread with 3.53% psyllium and 75% hydration would yield the best physical properties. Even though it was possible to find an optimum recipe for psyllium‐enriched bread, all rheology properties during dough development (maximum height of gaseous production, total gas volume, gas retained in the dough) were negatively impacted when psyllium was added—that is, 0% additions were superior. In another study, a 10% wheat flour replacement with psyllium increased slowly digestible starch and decreased rapidly digested starch when compared to control wheat bread, but bread enriched with psyllium had a lower volume and porosity, and increased hardness and chewiness (Bilgic and Sensoy 2023). Additionally, psyllium decreased hardness in wheat flour crackers—this is considered a negative effect as hardness in crackers could be related to fracturability and crispiness, which is desirable in crackers. One possible reason for these effects could be the fact that water addition was not adjusted when psyllium was incorporated. Raymundo et al. (2014) found that psyllium can be incorporated into wheat biscuits from 3% to 9%, but higher psyllium replacement (up to 15%) resulted in unworkable dough that could not be shaped. Psyllium (3%–9%) caused higher spread ratios, increased hardness, and lower cohesiveness, indicating that dough with psyllium affected internal cohesion, leading to altered texture. In a continuation of this work, Fradinho et al. (2015) found a similar trend: increasing psyllium in biscuit formulation negatively affected texture and color. The overall sensory acceptance was highest in biscuits with 3.9% psyllium and 51.4% wheat flour, but the optimal formulation determined by response surface methodology was 6% psyllium and 48% wheat flour.

Notably, as bakery products contain high amounts of starch, they can lead to blood glucose spikes, a known risk factor for metabolic syndrome. Some fibers are well‐known to reduce the GI of starchy foods, but there are many mechanisms, and this has not been well‐studied for *Plantago*. In one rare study, it was shown that psyllium addition to wheat, potato, or tapioca starch can reduce starch gelatinization (Sevilmis and Sensoy 2022); however, this effect is seen when psyllium addition is very high (psyllium:starch = 1:1). A 50% addition of psyllium to food is an unattainable and potentially hazardous quantity to incorporate into an actual food product. High intake of psyllium can lead to potential health issues. Namely, excessive psyllium consumption without adequate water intake can lead to constipation and, in some cases, bowel and gastrointestinal obstructions (Hefny et al. 2018; van der Schoot et al. 2022; Tominaga et al. 2023). These instances are reported when patients are taking psyllium as a supplement, typically in the form of husk or powder, rather than as a component of a food product. This is particularly noted in the elderly, people who are fasting or have fasted in a period before taking psyllium, or people with pre‐existing bowel abnormal structures. Garg (2017) has specifically highlighted the importance of sufficient water intake with psyllium as it has proven to be beneficial in clinical practice. He has recommended 20 g of fiber supplement per day with 500 mL of water at least (Garg 2017). However, more realistic (but still high) additions (17.14%) have seen large reductions in GI and glycemic load in vivo (Fratelli et al. 2018). The underlying mechanism is underexplored, and the influence of psyllium on starch digestibility properties, such as gelatinization degree, granule swelling, rupturing, and structure after thermal treatment, should be further explored.

Beyond baked goods, psyllium can be added to fermented dairy products as a fat replacer and to decrease syneresis (release of water from the food product during storage). When psyllium was incorporated into yoghurt at levels of 0.1%, 0.3%, 0.5%, and 0.7%, it was found that the highest addition (0.7%) resulted in the highest microbial count and ash content (Bhat et al. 2018). Flavor and textural attributes (hardness, gumminess, and adhesiveness) were negatively impacted by psyllium. However, yoghurt with 0.5% had the highest acceptance.

Psyllium‐enriched pectin gels (3 g/100 g), suitable as fiber‐rich jam models, showed no syneresis and high cohesiveness but were overly gummy with low fracturability and hardness (Figueroa and Genovese 2018). However, when in combination with other fibers (bamboo, apple, or wheat), psyllium gels showed desirable texture and stability. In a further study focused on apple jam (Figueroa and Genovese 2019), the authors again confirmed increased gumminess of the jam when psyllium was used alone. In combination with other fibers, it resulted in improved texture and mouthfeel, as well as stability up to 30 days.

In a study by Ferjančič et al. (2021), low‐fat chicken sausages were prepared with 3% or 6% psyllium husk addition to replace the missing fat component. As expected, enriched sausages had higher fiber content, though psyllium did not negatively affect texture properties. However, the color was significantly changed. In sensory evaluation, sausages with 3% psyllium addition were accepted by consumers, but sausages with 6% addition received lower scores, particularly regarding mouthfeel, juiciness, and appearance. Overall, psyllium has a positive influence on a variety of food products—it is a common improver of GF bread, and it could be implemented in meat replacement products, especially as their popularity is rising. However, psyllium can cause lower product quality when implemented in sufficient quantities to meet a “high in fiber” claim, which is 6 g of fiber per 100 g of product, or 3 g of fiber per 100 kcal (European Parliament 2006). Its high WAC and rheological properties represent certain technological challenges, which make it unsuitable for all applications. Oftentimes, positive effects are closely linked with certain negative traits. In the next section, the possible avenues for expanding *Plantago* mucilage use through natural or induced variation in chemical and physical properties are presented.

## 5 Strategies to Broaden *Plantago* Mucilage Functionality and Implementation

**FIGURE 4:**
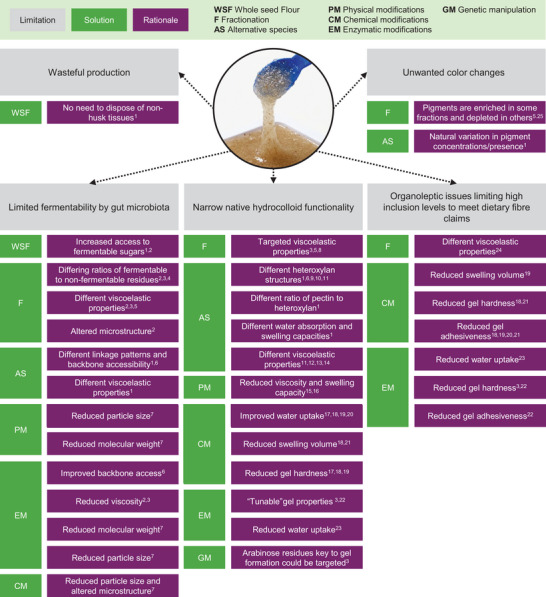
An overview of key limitations associated with implementation of psyllium husk in products. Strategies from Section 3 that may have promise as solutions to overcome these limitations are included along with a high‐level rationale. Specific precedents associated with the proposed rationale are included as superscript numbers: (1) Cowley et al. (2021); (2) Harris et al. (2023); (3) Marlett and Fischer (2003); (4) Yu et al. (2017); (5) Ren et al. (2020); (6) Phan et al. (2016); (7) Pollet et al. (2012); (8) P. Zhou et al. (2022); (9) Zhao et al. (2014); (10) Addoun et al. (2020); (11) Benaoun et al. (2017); (12) Yin et al. (2016); (13) Hesarinejad et al. (2018); (14) Behbahani et al. (2017); (15) Mallikarjunan et al. (2024); (16) Mallikarjunan et al. (2021); (17) Liu et al. (2010); (18) Niu et al. (2012); (19) Niu et al. (2013); (20) Cheng et al. (2009); (21) Liu et al. (2010); (22) Yu et al. (2003); (23) Yu et al. (2008); (24) Fradinho et al. (2020); (25) Niu et al. (2019).

Altogether, there is a rich literature highlighting the unique and commercially important properties of *P. ovata*‐derived psyllium husk and its mucilage. However, there is evidence in the literature that would suggest that there is little genetic variation among cultivated *P. ovata* accessions (Dhar et al. 2005; Kour et al. 2016) and, therefore, likely very little variation in functionality that can be exploited for targeted industrial use. Certainly, the many studies of *P. ovata* mucilage chemistry and rheology from researchers around the world yield remarkably similar results (covered in detail in Section 3). It is possible that psyllium's functionality could be improved or made more potent, reducing demand and tempering the fluctuations in price. It may further improve its properties as a dietary fiber. Figure 4 shows five common limitations of psyllium husk implementation in food products and the possible solutions that have been studied and are shown to be successful, with a brief description of the mechanisms through which those improvements are achieved. The specific precedents displayed in Figure 4 are described in detail in this section.

### 5.1 Use of Other *Plantago* Species

**FIGURE 5:**
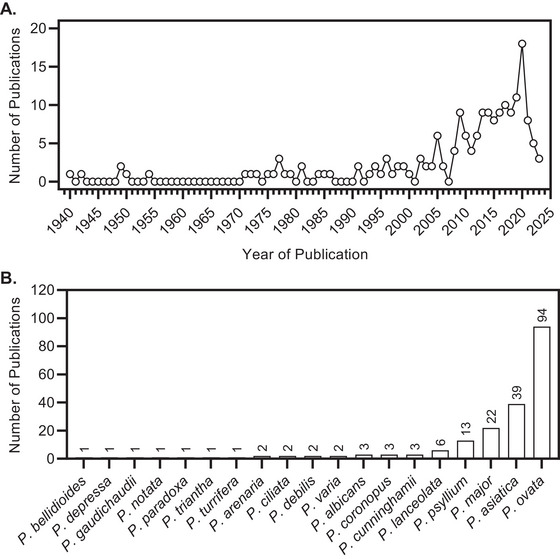
Bibliometric data of publications relating to *Plantago* seed polysaccharides between 1940 and 2023 (A), then delineated by species studied (B). The search engine Scopus was used with the term “TITLE‐ABS‐KEY (Plantago AND seed) AND polysaccharide OR mucilage OR hydrocolloid OR gum AND NOT (musa).” “*Musa*” was excluded to save screening and excluding large numbers of articles on starchy banana, as the common name “plantain” is shared by both *Musa* and *Plantago* species and often results are shared. Entries (*n* = 312) were manually screened to remove nonrelevant articles, and the species studied in articles deemed relevant (*n* = 19) were identified, with the number of publications in which they were studied recorded.

*Plantago ovata* is the only *Plantago* species utilized commercially for its seed and fiber, even though *at least* 77 of them produce mucilage (Cowley and Burton 2021). One reason for this is related to ease of processing and the anatomical nature of how the mucilage polysaccharides are stored in the mature seed: psyllium husk is a cell‐less layer of dried, laminated polysaccharides that can easily be milled from the seed surface. To our knowledge, only *P. ovata* (and perhaps closely aligned relatives) can be processed in this way, as other species have mucilage stored in discrete cells that are not easily separated from the seed (Cowley and Burton 2021). For this reason, *P. ovata* is the most extensively studied *Plantago* species despite underutilized relatives showing promise and recently becoming more studied (Figure 5). In fact, mucilage from non‐*P. ovata Plantago* species can be applied to products through extraction methods similar to those used for psyllium husk or the use of whole seeds and whole seed flour (WSF). Thus, the potential for exploiting natural variation in *Plantago* mucilage properties for tailored end uses is substantial.

In a comparative study of 12 *Plantago* species native to or naturalized in Australia, mucilage traits varied significantly between the studied species (Cowley et al. 2021). Mucilage yield ranged from 23.84% (*P. ovata*) to 4.43% (*P. paradoxa*), though most species were higher yielding (median = 15.52%, per seed weight). WAC was also tested, showing similarly wide variation: *Plantago turrifera* had the highest WAC (22.20 mg H_2_O/mg seed), and *Plantago triantha* had the lowest (1.42 mg H_2_O/mg seed). Interestingly, the relationship between yield and WAC is not perfectly linear. This is consistent with some earlier studies comparing *P. ovata* to its wild allies (Sharma and Koul 1986), suggesting that the chemical differences detected, which result in particular mucilage functionalities, are not explained entirely by polysaccharide concentration. In a follow‐up study applying flour from *Plantago* seeds to starch‐based food systems, it was found that the proportion of gel‐like fractions in the mucilage was more strongly correlated with water‐binding functions than abundance (Cowley et al. 2025). Several other studies have delved deeper into linking chemistry with functionality for mucilage polysaccharides from non‐*P. ovata Plantago* species.

*Plantago depressa* mucilage (extracted at 100°C) was divided into four fractions using chromatography (Zhao et al. 2014). All four fractions had high molecular mass (>1200 kDa) and were protein bound. Their composition included acidic polysaccharides with relatively high arabinose (up to 55.7%) and fucose (up to 28.7%) content, but low xylose (up to 11.6%), which is quite different from *P. ovata*. Fraction 3 had a distinctly high mannose content (28.1%). All fractions, and in particular Fraction 3, showed immunomodulatory effects, measured as lymphocyte proliferation, TNF‐α (tumor necrosis factor α) production, and NO (nitric oxide) content (Zhao et al. 2014).

*Plantago asiatica* alkaline‐extracted mucilage (water extractable fraction was discarded prior to alkaline extraction) was identified as a highly branched AX with 20.5% uronic acid and an A:X ratio of 1:4.1 (Yin et al. 2016). Rheologically, the mucilage displayed shear thinning behavior, depending on concentration and frequency, as well as thermal hysteresis (*G*ʹ = *G*ʺ) at 55°C. Based on current knowledge, thermal hysteresis is unique to this *Plantago* species. Interestingly, the alkaline fraction from *P. asiatica* was characterized as a weak gel, unlike the AEF from *P. ovata*, which is described as a strong gel. When Ca^2+^ and Na^+^ were added, *G*ʹ and crossover temperature were increased. Moreover, Ca^2+^ gel showed lower frequency dependence, higher *G*ʹ, and superimposed heating and cooling curves. Therefore, its behavior was more representative of a true gel.

Behbahani et al. (2017) have used response surface methodology to determine the optimal conditions for the aqueous extraction of mucilage from *Plantago major*, which were 75°C, pH 6.8, and a seed‐to‐water ratio of 1:60. These conditions yielded 15.18% extracted polysaccharide, with a molecular weight of 1200 kDa, that displayed high total phenolic and flavonoid contents (76.79 mg GAE/g and 97.8 mg/g, respectively) and could be used as a powerful antioxidant in food products. The extract showed applicability as an emulsification agent and foam stabilizer (67.4% emulsion stability and 88.4% foam stability).

*Plantago ciliata* polysaccharides were aqueously extracted (at 60°C) with a yield of 18.6% (Addoun et al. 2020). They were characterized as HXs with an A:X ratio of 1:4 and a molecular weight of 700 kDa. The rheological properties were described as those of a weak gel, both in water and salt solutions. When considering health properties, *P. ciliata* showed antihyperglycemic and anti‐inflammatory effects (Addoun et al. 2021). The extract inhibited the activity of α‐amylase, presumably due to fiber enveloping starch granules, thereby blocking α‐amylase access. Moreover, it showed the inhibition of α‐amyloglucosidase, possibly due to its highly branched structure.

A water extractable (60°C) fraction of *Plantago notata* was characterized as HX with a predominant composition of xylose, rhamnose, and arabinose (77.4, 9.20, and 7.58 mol%, respectively) and a molecular weight of 2300 kDa (Benaoun et al. 2017). Rheologically, the fraction can be characterized as a weak gel. Interestingly, the authors have described the xylan backbone as having a combination of both β‐(1,4) and β‐(1,3) linkages. While a mixed‐linkage xylan from *Plantago* has been proposed before, the current scientific consensus is that β‐(1,3) linkages exist in unusual side chains rather than the backbone (Cowley and Burton 2021).

*Plantago lanceolata* seed mucilage solution was characterized as a weak gel with slight frequency dependence (Hesarinejad et al. 2018). When increasing the concentration (0.5%–2%), complex viscosity increased, which points to the usefulness of *P. lanceolata* mucilage as a food thickener or stabilizer. Heating and cooling cycles showed hysteresis, but it decreased with lower heating‐cooling rates, pointing to the formation of hydrogen bonds and a gel network. In this work (Hesarinejad et al. 2018), polysaccharides from *P. lanceolata* were aqueously extracted, presumably at room temperature for 1 h (temperature was not specified). Monosaccharide composition primarily consisted of mannose (69.42%), arabinose (11.98%), glucuronic acid (7.45%), and xylose (7.42%), and as such, the polysaccharide is described as an “arabinoxylomannan.” However, when compared to other published results (Cowley et al. 2021, and new data still not published), xylose is the dominant monosaccharide in *P. lanceolata* gel. A possible explanation could be mannose leaching from endosperm during the extraction process, which has been observed previously (Cowley et al. 2020), but considering that the extraction was not long (1 h) or harsh (room temperature), this is most likely not the case. A likely explanation could be monosaccharide analysis itself—HPAEC‐PAD was used to determine sugar composition, and the overlap of xylose and mannose is a widely known occurrence in this system (Nagel et al. 2014). It is likely that the reported high amount of mannose is actually xylose. *Plantago lanceolata* gel composition should be confirmed, but rheological data still suggest that its application in food systems is a viable option.

### 5.2 Use of Fractionated *Plantago* Mucilage

As shown clearly for *P. ovata*, mucilage fraction chemistry and functionality are influenced by extraction protocols (Section 2). Studies that have prepared specific fractions of *Plantago* mucilage using methods like temperature fractionation are discussed. Therefore, using specific fractions (instead of whole husk) could be a useful option to overcome the challenges described previously.

In a study of 12 diverse *Plantago* species (Cowley et al. 2021), mucilage was fractionated by a simple method employing temperature and agitation (CWE = cold water extract, HWE = hot water extract, IAE = intense agitation extract). The highest pectin‐to‐HX ratio was found in CWE of *Plantago bellidioides*, followed by *Plantago coronopus* (1.99:1 and 1.40:1, respectively). On the other hand, *P. major* and *P. lanceolata* showed low pectin components (0.23:1 and 0.34:1, respectively). A:X ratio is an important factor in assessing AX properties, as it indicates the degree of branching, which relates to extractability and susceptibility to enzymatic hydrolysis. The trend shows an increase in A:X ratio with further extraction steps in almost all species, consistent with previous literature (Cowley et al. 2021), although more sophisticated chemical analyses are needed. Moreover, it was found that the sum of HWF and IAE yield strongly correlates with WAC; however, content does not perfectly explain all of the differences in WAC, so more research is needed linking chemistry with this property.

Niu et al. (2019) have studied interactions and functionality of whey protein and psyllium (1:2) prepared at different temperatures (room temperature, 60°C, and 90°C). Treatment at 90°C resulted in a more uniform network, but it increased hardness, gumminess, chewiness, and resilience when compared to mixtures treated at 60°C. The authors explained this as higher protein denaturation at 90°C—exposed amino groups of protein could lead to pronounced carbonyl ammonia reaction, which favors homogeneous structuring and, therefore, increased hardness. However, according to the overall rheology profiles of fractionated *P. ovata* mucilage discussed in Section 3, higher extraction temperatures result in higher storage moduli (which relates to the hardness of a material). Thus, such a texture profile could instead easily be a consequence of the different temperature treatment of the psyllium fiber. Texture of the psyllium fractions alone was not shown. Nevertheless, psyllium–protein interactions, especially using a wider range of fractions, should be the subject of future research.

Fractionated psyllium gel can be added to GF pasta as a structural network component (Fradinho et al. 2020). When comparing the best processing conditions of psyllium, it was determined that psyllium husk (160–315 µm particle size) treated at 40°C resulted in pasta with desirable properties. Assessed temperature treatments showed that a 20°C gel did not reach full maturation, while the others (40°C, 60°C, 80°C, 90°C) did, and they showed weak gel‐like structures and lower firmness. However, pasta incorporating a 40°C gel had lower adhesiveness (which is a negative trait in pasta). Overall, the pasta that incorporated psyllium gel and rice flour (50:50) had lower cooking loss and reduced carbohydrate digestibility. Possibly, the gel treated at 40°C could provide more structural integrity than the gel treated at 20°C, but also had desirable water holding and gel properties, which are lower than those of gels treated at higher temperatures.

The interaction of fibrillated cellulose (FC) and psyllium (PSY) HX fractions has been explored by Ren et al. (2020)(). It was shown that FC–PSY composites have potential for incorporation into food products to obtain targeted rheological properties. Fluorescent microscopy showed that the psyllium fraction extracted at 60°C (PSY‐60) was affixed to FC, while other fractions (PSY extracted at 20°C, 60°C, and 80°C, and whole psyllium husk) did not show association with FC. The authors attributed this phenomenon to the highly branched structure of PSY‐60, which could have stereochemical compatibility with FC, allowing interactions by hydrogen bonding.

Fiber from fractionated *Plantago* mucilage has found applicability in emulsion fabrication. The self‐healing ability of HW (85°C)‐extracted *P. ovata* mucilage was found to be a key factor in oil‐in‐water emulsion stability (Zhou et al. 2022). When used above its gelling point (0.5%), *P. ovata* polysaccharides formed networks in which oil droplets were embedded, and which could survive high shear during emulsion preparation. Works published by Niknam et al. (2018, 2020) have shown the applicability of ultrasound/HW‐extracted *P. major* polysaccharides (75°C, 40 min) as an emulsion and foaming stabilizer. The addition of *P. major* polysaccharides (0.3%–1%) increased emulsion storage stability, enhanced heat and freeze–thaw stability, and decreased creaming (separation of emulsion phases). Foaming capacity decreased, but foaming stability increased with higher polysaccharide content.

Studying the fermentability of different *P. ovata* fractions, as opposed to whole psyllium husk, has given valuable insights that might elucidate possible answers to seemingly contrasting results. Marlett and Fischer (2003) have described three isolated psyllium mucilage fractions: A—alkali insoluble, B—alkali insoluble and acid soluble, and C—alkali and acid soluble. Fraction A had the highest amounts of arabinose and was not fermentable. Fraction B (55% of psyllium mucilage) formed gels, was poorly fermented, and was the fraction responsible for increases in stool moisture and bile acid extraction. Fraction C (<15% of psyllium mucilage) is viscous and rapidly fermented. The authors attributed differences in fermentability to different structural properties and gel‐forming potential of fractions, as well as “yet unidentified structural features that hinder fermentation by typical colonic microflora.” Recently, Harris et al. (2023) have compared the fermentability of three *P. ovata* mucilage fractions (F1—CW extractable fraction, F2—HW extractable fraction, and F3—alkali extractable fraction) in in vitro conditions. The fractions show specific rheological profiles—F1, a viscoelastic fluid; F2, a weak gel; and F3, a true gel. F1 had higher amounts of galacturonic acid and rhamnose, while F2 and F3 had virtually identical monosaccharide and linkage composition. However, it should be noted that the hydrated psyllium fractions (0.5 g in 81 mL serum) were homogenized with an Ultra‐Turrax for 3 min to allow for sampling, which could have reduced the gelling capacity. This allows an understanding of how gelling affects fermentability while keeping other variables (mainly linkage, which is known to influence fermentability) consistent, but it may not reflect the in vivo process. Moreover, this could provide answers for some of the inconsistent findings in earlier fermentation studies about psyllium. The highest total gas was produced by F1 (34.27 mL) and the lowest by F3 (3.00 mL). This indicates that the low viscosity of F1 enabled the intestinal enzymes to reach the substrate. The main metabolite of all three fractions was acetate, followed by propionate and butyrate (approximate ratio for fractions and husk was 57:36:7). Interestingly, F2 and F3 had a very similar metabolite profile. The authors concluded that gas production is related to viscosity, while linkage composition and side chain motif affect SCFA output.

Altogether, using fractions of psyllium will affect product quality in different ways. A simple CW and HW extraction will yield fibers with different properties. For example, one could choose CW‐extracted psyllium when trying to achieve a food structure that is more viscous and less gel like (such as beverages, sauces, and similar), or when a lower WAC is beneficial for texture (such as bread). The higher fermentability of CW‐extracted psyllium compared to raw psyllium and other fractions may also be desirable in products. A HW fraction could be used when fiber type is important (i.e., when one would want only HX, without a pectin component) or when trying to achieve “solid” gel structure, perhaps in jellies or set yoghurts. The development and optimization of tailor‐made blends of fractions for targeted properties could be a rich area for future studies. Finally, fractionation offers opportunities to use “clean” fibers that are not modified or treated, as such additives may have negative consumer perceptions (Kajzer and Diowksz 2021).

### 5.3 Whole seed flour

*Plantago ovata* is mostly used for husk production, with 75% of seed yield not being utilized, thus making its production hugely wasteful (Cowley et al. 2021). *Plantago ovata* seed composition is shown to be highly nutritious and less viscous. For this reason, whole seed flour (WSF) could be used to improve the nutritional and functional profile of food products.

Noguerol, Larrea et al. (2022) have compared a wide range of physicochemical properties of *P. ovata* husk (*Plantago* husk, PH) and *P. ovata* WSF (*Plantago* powder, PP). When compared to PH, PP had higher protein (2.5% vs 6.55%), lipid (0.5% vs 1.8%), mineral (2.1% vs 3.1%), and soluble fiber content (0.9% vs 42.1%). Moreover, PP had higher total phenolic content and antioxidant activity (900 mg GAE/100 g and 126 mg TE/100 g, as compared to PH, which had 55 mg GAE/100 g phenolic content and 52 mg TE/100 g antioxidant activity). As expected, PH had higher water holding capacity and water retention capacity, while PP was more soluble. This indicates that PP could be easier to implement in food products. However, PP had a darker color at all tested concentrations (1%–7%), and the heating treatment (at 65°C) resulted in an increase in darkness, redness, and yellowness (Noguerol, Larrea et al. 2022). Overall, it seems that PP (from whole seeds) provides nutritional benefits; however, both samples had certain functional advantages and disadvantages.

By leveraging both natural variation in mucilage properties and the use of WSF, these advantages and disadvantages can be balanced. By applying WSF from several species from Australia with large differences in mucilage chemistry and functionality, a wide range of GF bread qualities was possible to produce (Cowley et al. 2025). Several WSF types produced GF bread of comparable quality to commercial *P. ovata*, though some had improved color, texture, and crumb structure. Species like *P. turrifera* may therefore have more commercial potential if the use of WSF is preferred due to its improved quality in GF bread, minimal color impacts, and agronomic advantages (Herliana et al. 2025).

WSF from *P. ovata* and *P. psyllium* was compared with psyllium husk substitution (4% and 8%) in wheat bread (Pejcz et al. 2018). Psyllium husk substitution at both levels increased crumb moisture and volume, while WSF from *P. psyllium* had a negative effect on bread volume. Incorporation of both WSFs as well as husk led to a darker crust color, but there was no significant difference from the control in overall sensory acceptability. Bread with WSF had higher protein content, while bread with husk had higher dietary fiber content (Pejcz et al. 2018). Psyllium husk is a common addition in GF breads, as it can improve their nutritional and technofunctional properties (Belorio and Gómez 2022). WSF was incorporated into GF rice bread—5% of flour was replaced by either whole or ground *P. ovata* or *P. psyllium* seeds (Ziemichód et al. 2019). Replacement with whole *P. ovata* and both types of *P. psyllium* seeds increased specific volume, but ground *P. ovata* seeds resulted in bread with a specific volume lower than that of the control. Both seed types had a protective effect on storage properties (hardness), with ground seeds showing lower hardness, which is desirable.

When psyllium husk (PH), psyllium husk powder (PW), and *P. ovata* seeds powder (PP) were incorporated into plant‐based sausages (3%–6%), it was determined that fiber addition resulted in improved nutritional profile (decreased fat content and increased ash and carbohydrate content) (Noguerol, Igual et al. 2022). Texture profile analysis has shown increased hardness when all fibers were added, but the lowest increase was in PP sausages. Water release was also the lowest in PP sausages, which is an important parameter for their quality and acceptance. However, sensory evaluation showed the highest overall acceptability of plant‐based sausages with PW (5 out of 7 score, whereas the control had a score of 3). Moreover, color changes were the least prominent in PW sausages, while PP sausages had a negative color change and were evaluated the lowest on visual and color score when compared to PH and PW sausages (Noguerol, Igual et al. 2022).

Detailed nutritional profile of WSF of 12 *Plantago* species, including *P. ovata*, has shown the potential to enrich foods with protein and unsaturated fatty acids, such as omega‐3 and omega‐6 (Cowley et al. 2021). *Plantago paradoxa* and *P. triantha* had >30% protein content, which is higher than that of chickpeas. A heart‐healthy ratio of omega‐3 to omega‐6 for cardiovascular health is recommended to be 4:1 (Simopoulos 2002); *P. turrifera* has shown the said ratio to be 4.85:1, which is higher than flaxseed. Additionally, WSF from *Plantago* seeds does not contain starch but contains mannans and fermentable oligosaccharides, which were hypothesized to give WSF fermentative benefits over husk (Cowley et al. 2021). Indeed, when compared with husk in an in vitro fermentation study, WSF was better fermented than husk, with higher gas production and butyrate content and lower propionate content (Harris et al. 2023).

### 5.4 Modifications

A book chapter by Yu et al. (2009) was published on the health benefits of psyllium and approaches to improve its functionality, including changing the pH, hydrothermal treatments, conventional enzymatic treatments, solid‐state enzymatic modifications, and chemical changes, such as grafting, crosslinking, and alkaline treatments. Therefore, this section is an overview of psyllium modification papers published after 2009. In the said book chapter, the authors described different modification types used for psyllium and highlighted the usefulness of solid‐state enzymatic reactions (Yu et al. 2003). In general, modifications reduced WAC and swelling capacity of psyllium while maintaining or improving certain health benefits. This poses a question: Is high viscosity or the unique structure of psyllium responsible for physiological effects, or can these two aspects be observed independently? The papers included below show that modifications can have different overall results, which can be explored for greater benefits and ease of application. However, as the observed effects were discovered using in vitro assays, in vivo studies are necessary. Considering that health effects in people are explained mostly by high viscosity (Section 2), it is worth examining how psyllium with reduced viscosity would perform.

Modifications aimed at altering the functionality of *Plantago* species in health and food applications are listed in Table 1, although there are more describing pharmaceutical and engineering uses.

**TABLE 1:** Modifications applied on *Plantago* polysaccharides and subsequent results. Table includes type of modification, brief explanation of protocols, and main results from papers on *Plantago* fiber transformation published after 2009.

Type of modification	Polysaccharide source and modification protocol	Results
Physical—gamma radiation (Mallikarjunan et al. 2021)	Psyllium husk processed by gamma radiation at 5, 15, 25, and 50 kGy	Solubility was increased proportionally to radiation dose Molecular weight, swelling capacity, and gel strength were decreased Bile acid and glucose retardation index were lower for irradiated psyllium than untreated Control *dhokla* (Indian snack food) had higher sensory acceptability than *dhokla* fortified with treated psyllium, but textural parameters were not significantly different In vitro starch digestibility was lower for fortified *dhokla*
Physical—gamma radiation (Mallikarjunan et al. 2024)	Psyllium husk powder processed by dry state gamma radiation and 25‐kGy dose was chosen	Irradiated psyllium formed weaker gel than nontreated psyllium Increase in water uptake capacity and dough weight of *chapati* was observed—increase was higher in irradiated psyllium due to higher concentration of fiber used (14% for irradiated vs. 4% for nonirradiated psyllium) *Chapati* with psyllium had lower hardness after baking and after 24 h of storage *Chapati* without psyllium and with nonmodified psyllium had medium glycemic index (68 and 63, respectively); *chapati* with irradiated psyllium had low GI (46)
Chemical—acidic (Cheng et al. 2009)	Psyllium husk was acid hydrolyzed by HCl (0.36%, 0.72%, 1.44%, 2.88%, 4.5%) for 5 days	Acid‐treated psyllium had lower water uptake properties, as well as gel adhesiveness and hardness 0.36% HCl‐treated psyllium did not lose its swelling capacity (measured with simulated intestinal fluid), but others did—psyllium treated with up to 1.44% HCl was dose dependent, but 2.88% and 4.5% HCl treatments were time dependent as well Psyllium treated with up to 1.44% HCl did not lose the ability to bind bile acids when compared to untreated psyllium, but higher concentrations led to loss
Chemical—sulfation (Liu, Zhang, et al. 2010)	Acid‐treated psyllium husk was sulfated by sulfur trioxide‐pyridine agent in dimethyl formamide to obtain three different sulfated derivatives (SP1, SP2, SP3)	Elemental analysis showed sulfur content (%): SP1—10.37, SP2—11.11, SP3—13.11 Sulfated psyllium showed decrease in gel strength (*G*ʹ) and lower apparent viscosity (proportional to sulfation degree) Sulfated psyllium had higher water uptake capacity, but swelling volume (measured with simulated intestinal fluid) of SP1 was lower, while SP2 had same swelling volume as the control Sulfated psyllium had higher bile binding capacity than the control—SP1 had 6.5×, SP2 had 5.9×, and SP3 had 6.3× higher cholic acid binding capacity than the control
Chemical—hydroxypropylation (Liu et al. 2010)	Acid‐treated psyllium husk was mixed with isopropanol and sodium hydroxide, and then, different amounts of propylene oxide were added to produce four different hydroxypropyl psyllium derivatives (HP1, HP2, HP3, HP4)	Hydroxypropyl content (%): HP1—6.33, HP2—10.47, HP3—12.63, HP4—15.26 Hydroxypropylation reduced gelling capabilities of psyllium—HP gels had lower hardness and adhesiveness Swelling volume (measured with simulated intestinal fluid) of HP1 was the highest, while HP2 was not different than the control, and HP3 and HP4 had the lowest swelling volume Water uptake capacity of all HP derivatives was not significantly different than the control Binding capacity for cholic acid was the highest in HP1, while HP2 was not significantly different from control and untreated psyllium, and HP3 and HP4 had the lowest capacity Binding capacity for chenodeoxycholic acid was statistically same for untreated psyllium, control, HP1, and HP2, while HP3 and HP4 had lower capacities
Chemical—succinylation (Niu et al. 2012)	Psyllium was succinylated with three different doses of succinic anhydride and tributylamine to create three succinyl psyllium derivatives with low, middle, and high levels of substitution	Succinyl substitution degree: low—1.9, medium—3.3, high—3.9 Succinylation reduced gel strength and adhesiveness in dose‐dependent manner Water uptake capacity was increased, but swelling volume (measured with simulated intestinal fluid) was decreased when compared to native psyllium Water uptake capacity and swelling volume were inversely correlated to the degree of succinylation Succinylated psyllium had higher bile acid binding capacity than native psyllium, but they were not significantly different from each other
Chemical—sulfation, Hydroxypropylation, succinylation (Niu et al. 2013)	Methods described in three previous rows	Degrees of substitution: sulfated psyllium (SFP)—1.02, hydroxypropylated psyllium (HP)—0.88, succinylated psyllium (SCP)—0.79 All three derivatives had lower gel hardness and adhesiveness than untreated psyllium Among modified psyllium, SFP had highest values of gel hardness and adhesiveness, while there was no significant difference between HP and SCP SFP and SCP had significantly higher water uptake capacity, while HP did not differ from control psyllium Swelling volume (measured with simulated intestinal fluid) was reduced when chemical modifications were applied—HP had the lowest swelling volume, followed by SCP and SFP Bile acid binding capacity: SFP > SCP > HP > psyllium
Biological—partial hydrolysis with Viscozyme L (Yadav et al. 2016)	Psyllium husk was partially hydrolyzed by Viscozyme L (cellulase, hemicellulase, xylanase, arabinose, β‐glucanase activities) in solid‐state reaction; yoghurt was supplemented with native and modified psyllium, and Wistar rats were fed for 90 days	Blood lipid profile (triglycerides, total cholesterol, HDL, LDL, VLDL, atherogenic index) measured after 90 days showed significant differences in rats fed with native and partially hydrolyzed psyllium when compared to rats fed with conventionally prepared yoghurt Blood lipid profile (triglycerides, total cholesterol, HDL, LDL, atherogenic index) measured after 90 days did not show significant differences between native and partially hydrolyzed psyllium, except in VLDL level, which was higher in native psyllium group Rats fed with partially hydrolyzed psyllium had a higher SCFA count than rats fed with native psyllium, and both of them had higher count than other groups Higher lactobacilli and lower coliform count were found in rats fed with modified psyllium
Combined—ball milling and different extraction protocols (Pollet et al. 2012)	Psyllium husk was ball milled for 48 h (500 rpm) and further extracted using four protocols (different solvents, temperature, and pH) to get four fractions with specific DP and A:X ratios	DP from highest to lowest was: 300, 200, 88, and 72 A:X ratio from highest to lowest was: 0.29, 0.27, 0.16, and 0.14 Fraction with highest DP (300) was least fermentable (around 30%) Fermentation with lowest DP was the most fermentable (50%) Fermentation of fractions with DP of 200, 88, and 72 resulted in highest acetic and propionic acid concentrations Fermentation of fractions with DP of 300, 200, and 88 resulted in highest butyric acid concentration

#### 5.4.1 Physical Modifications

Psyllium husk was processed by gamma radiation and used to make *dhokla* (Indian snack food) (Mallikarjunan et al. 2021). The husk was first extracted as a water extractable and alkali extractable fraction (AEG), and it was reported that solubilization of AEG by gamma irradiation at different levels (5, 15, and 25 kGy) was the contributing factor to overall husk solubilization. It was shown that *dhokla* fortified with processed psyllium husk received better sensory analysis scores than *dhokla* with native psyllium, which was described as gummy and chewy. Moreover, irradiated psyllium lowered sugar release in in vitro digestion when compared to unfortified *dhokla*, showing potential for developing foods with reduced GI. A positive effect of gamma irradiation on psyllium powder was reported for *chapati*, Indian unleavened bread (Mallikarjunan et al. 2024). When irradiated, 14% psyllium incorporation in *chapati* was possible without negatively impacting its physicochemical and sensory properties, while untreated psyllium was incorporated only up to 4%. *Chapati* with modified psyllium (14%) had a low GI, while nonfortified and *chapati* fortified with native psyllium had a medium GI. Irradiated psyllium had higher solubility, which was caused by breakage of glycosidic bonds and chain scission (degradation of polymer main chain, which is a common occurrence in polymer radiation).

Polysaccharides from one of *P. ovata*’s relatives, *P. asiatica*, have been modified by high‐pressure homogenization (HPH) (Hu, Nie, and Xie 2013), microwave irradiation (Hu, Nie, Li, et al. 2013), and ultrasound (Huang et al. 2018). These processing methods have shown changes in polysaccharide structure and functionality, increased fermentability (Hu, Nie, Li, et al. 2013; Hu, Nie, and Xie 2013), and increased anti‐inflammatory activity (Huang et al. 2018) and thus could be viable options for modifying *P. ovata* fiber.

#### 5.4.2 Chemical Modifications

Using mild acid hydrolysis, psyllium husk was modified by HCl at different concentrations, and it was determined that HCl up to 1.44% can reduce the hardness and adhesiveness of psyllium gel without affecting its functional properties, such as bile acid binding (Cheng et al. 2009). This shows that mild acid treatment might be a promising candidate to change certain functional properties of psyllium, maintaining critical ones, while keeping the process inexpensive.

Chemical modifications, such as sulfation, hydroxypropylation, and succinylation, have been employed to improve the functionality of psyllium. Sulfation of psyllium resulted in a weaker gel structure and lower swelling capacity but higher water uptake and bile acid‐binding properties (Liu, Zhang, et al. 2010). SEM images showed changed structures after sulfation, which appeared grainy, possibly with a reduced surface area, unlike untreated psyllium, which was smooth. This change, as well as the introduction of negatively charged sulfonic groups, could have contributed to changes in the examined properties. Hydroxypropylation of psyllium husk revealed interesting results—two derivatives with a lower degree of hydroxypropylation (6.33% and 10.47%) had enhanced bile acid‐binding properties and swelling capacities, while derivatives with higher substitution (12.63% and 15.26%) had the lowest values of said properties (Liu, Xie, et al. 2010). In all cases, gel strength was reduced. Considering that the structure of all derivatives was similar, the authors concluded that the degree of substitution may have impacted binding capabilities. Succinylation of psyllium husk resulted in weaker gels, higher water uptake, and lower swelling capacity when compared to psyllium gel (Niu et al. 2012). Moreover, bile acid binding was increased in succinylated psyllium. Interestingly, three succinyl psyllium derivatives (with different substitution degrees—1.9, 3.3, and 3.9) were not significantly different from each other when observing water uptake or bile acid‐binding capacity. Finally, the same research group has compared all three chemical modification methods (Niu et al. 2013). The important conclusion of these comparisons was that the introduction of negative charges, size, and the steric effect of substitutions have different effects on the functional properties of psyllium. Charge influences bile acid‐binding (sulfated psyllium had the highest capacity) and water‐uptake capacity (sulfated and succinylated psyllium had the highest water uptake values). Altogether, the size of substitution groups could be the key to optimizing gel‐forming ability and swelling volume in chemically modified psyllium.

A worthwhile observation on chemical modifications is considering if changed psyllium would be acceptable to consumers, who appreciate “clean labeled” food products (Chen et al. 2022). It is plausible that chemically modified psyllium would be scrutinized in the food market as modified celluloses and starches are, thus questioning its applicability beyond research.

#### 5.4.3 Enzymatic Modifications

Psyllium husk, partially hydrolyzed by enzymes, was added to yoghurt before feeding it to Wistar rats for 90 days (Yadav et al. 2016). Both native and partially hydrolyzed psyllium husk showed better lipid profiles in blood plasma than conventionally prepared yoghurt and control groups, which shows that partial hydrolysis did not negatively impact the hypocholesterolemic effect of psyllium. However, very‐low‐density lipoprotein levels were higher for native psyllium. Furthermore, the prebiotic effect was enhanced when partial hydrolysis was applied to psyllium, as SCFA content and lactobacilli count were higher in rats fed with modified psyllium.

#### 5.4.4 Combined Modifications

Psyllium husk was ball milled and then extracted by four protocols to obtain arabinoxylan oligosaccharides (AXOSs) with different degrees of polymerization (DP) and A:X ratios (Pollet et al. 2012). For the first step of modification, extensive ball milling was conducted for 48 h. After this, the ball‐milled fiber was extracted under different pH conditions and temperatures. In vitro fermentation showed that all AXOS preparations were fermentable, and it was noted that long ball milling increased the fermentability of psyllium husk. AXOSs with the lowest DP and A:X ratio showed the highest degree of fermentation. Enzyme activity of arabinofuranosidases, xylanase, and xylosidase was increased, along with acetic, propionic, and butyric acid concentrations. It was suggested that this combined treatment can result in physiologically active AXOSs and was recommended for use in psyllium husk modification, where high WAC can present a challenge in food and health.

### 5.5 Genetic Strategies

The genetics of xylan synthesis are difficult to study, but the accessibility of psyllium's HX and synthetic tissue has made it useful as a model system to discover the underlying genes. A plethora of studies have implicated proteins in the GLYCOSYLTRANSFERASE (GT) families 43 and 47 in the elongation of the xylan backbone and GT61 proteins in the attachment of side chains (Ye and Zhong 2022). Supporting these, evidence from Expressed Sequence Tags and qPCR of developing psyllium integuments (containing the developing mucilage secretory cells) supports the roles of GT43, GT47, and GT61 in psyllium seed mucilage HX synthesis (Jensen et al. 2013; Jensen et al. 2014; Phan et al. 2016). Furthermore, suppression of GT43 and GT47 expression reduces xylan abundance (Lovegrove et al. 2013) and viscosity in wheat, while several GT61s are responsible for adding substitutions in rice (Zhong et al. 2021), likely working in tandem, suggesting that xylan patterning and properties could be manipulated in *Plantago* by targeting these gene families. The criticality of side chains in the functional properties of psyllium makes GT61s an appropriate target. Overexpression or suppression of modifying enzymes in the mucilage secretory cells during HX synthesis is another possible strategy demonstrated recently in *Arabidopsis* (McGee et al. 2021). The recent genome sequencing of two *Plantago* species (*P. ovata* and *P. major*) (Herliana et al. 2023; Lyu et al. 2023), the emerging techniques for the stable transformation of *Plantago* species (Levengood et al. 2023), and the changing legislation on genetic engineering and modification in foods make the future of a genetic strategy for altering psyllium's functionality a promising one.

## 6 Conclusions and Future Directions

In this review, a comprehensive summary of the health benefits, mechanisms of action, and food industry uses of psyllium husk is presented. Psyllium addition is widespread in food products, fueled by the aforementioned positive health effects, but certain challenges are evident.

First, the inconsistent naming of *P. ovata* in scientific and popular literature should be mentioned. Husk from *P. ovata* is certainly the most commonly used in research and in food product development and preparation. The *P. ovata* husk is well known as “psyllium husk,” but sometimes confusingly is simplified to “psyllium.” This oversimplification leads to confusion in the literature as it was derived from the common name “psyllium,” which is used for multiple *Plantago* species, not just *P. ovata*. A great example is the plethora of literature concerning “*Plantago psyllium* husk” or similar variations: *P. psyllium* (syn. *P. afra*) is a separate species from *P. ovata*, which has also been historically used as a supplement but cannot be husked in the same way (unpublished data). This literature is almost always referring to husk from *P. ovata*. Therefore, researchers must do their due diligence to provide a clear and detailed description of the materials used, including the scientific name of the plant, specifying the part used (husk or the whole seed), and steps of preparation (e.g., grinding). Specifically, if mucilage is used, then all conditions of extraction(s) should be clearly stated, and it should be known if any insoluble parts of the husk are kept or discarded. The “psyllium” is an established expression used in commercial settings and is unlikely to be changed, but it is recommended that researchers endeavor to verify the botanical source of their materials (using a botanist or verified by the supplier) and include this in detail in the materials and methods to ensure future reproducibility and easier interpretation of the literature. *Plantago ovata* husk, psyllium (*P. ovata*) husk, and even psyllium husk from *P. ovata* are sufficiently detailed alternatives to psyllium or psyllium husk alone.

Second, research regarding the long‐term use of food products enriched with psyllium is lacking. Regarding psyllium application in various food products, studies are mostly focused on products’ development, characterization, analysis, and sensory acceptance. While those areas are fundamentally important to achieve a stable food product with high consumer appeal, clinical studies including said products are needed as well. Given current trends around fiber intake and GF diets, we believe this topic is anticipated to become a major area of scientific and practical importance.

Through detailed structural characterization and rheological profiling, psyllium husk's high viscosity and gelling capabilities are associated with several positive properties, but their successful implementation is likely to be closely linked to its application challenges.

Therefore, possible scenarios for broadening the industrial use of psyllium are proposed:

1. The use of other *Plantago* species presents a high chance to obtain subtle but discretely different polysaccharides that might act as a toolbox for targeted functionalities without the need for modification. Limited but promising rheology studies have shown similar but distinct functionalities that show promise to this end.
2. The use of fractionation might allow targeted isolation of fractions with specific properties. These fractions are extremely useful in research, and there is increased interest in their incorporation as food and health additions, but prolonged time and energy‐intensive isolation processes might hinder the transfer of the promising research in this area to commercial scales. Streamlining and upscaling of fractionation processes at pilot plant scale for commercial product development ventures are strongly recommended.
3. WSF provides nutritional benefits, such as increased protein content and unsaturated fatty acids, as well as easier application in food products due to lower viscosity. In‐depth studies on WSF should be carried out, with a focus on fermentability because of the mannan‐ and oligosaccharide‐rich endosperm.
4. A wide range of modifications, including physical, enzymatic, chemical, and combined, offer opportunities to change the structure of psyllium and influence its functional properties, namely water absorption capacity and gelling behavior. By introducing different functional groups, it is possible to change the properties of the fiber or choose enzyme(s) that would result in fibers with a different structure. Importantly, modification‐associated decreases in gelling might allow greater additions in food products, but the evidence suggests that the dietary fiber potency would be affected when gelling is altered. This would affect the accuracy of dietary fiber claims associated with recommended psyllium doses. More research should be conducted to assess the dose dependence of dietary fiber benefits of modified psyllium and whether combinations might offer one practical solution to “meet in the middle.”
5. Genetic manipulation could be exploited to target backbone patterning of psyllium fiber; thus, the distribution and length of side chains, which are the main driver of gelling capacity, could be influenced. Additionally, enzymes encoded by *P. ovata*’s own genome might also offer tools for an enzyme biotechnology approach where existing xylanase enzymes are ineffective at digesting psyllium HX.
6. The inherent trade‐off between health effects and desirable sensory properties must be considered. For example, minimizing viscosity and swelling to maximize inclusion levels in products (e.g., by chemical modification) may negate the health benefits associated with these properties. Using modifications in combination, perhaps through sophisticated experimental design strategies, might help maximize the benefits of both aspects. Therefore, strategies and combinations of strategies should be compared to untreated psyllium husk as a minimum benchmark in both health and food research moving forward.

Combined, bridging of the gap between relating chemistry and functionality of fractionated *Plantago* mucilage, recent research on understudied relatives of *P. ovata*, and newfound knowledge provided by *Plantago* genomics present possible solutions to challenges arising when applying highly viscous mucilage into food products, as a health supplement, and in any other nonfood applications that take advantage of psyllium's unique properties. Success of these strategies is likely to be closely linked to their challenges, and the inherent trade‐off between the two must be considered. The utility of this review lies not only in identifying opportunities for targeted *Plantago* mucilage extraction and use but also in providing potential new approaches when researching other myxospermous species.

## Nomenclature

A:X: arabinose to xylose
AX: arabinoxylan
AXOS: arabinoxylan oligosaccharide
AEF: alkaline‐extracted fraction
CWE/CWF: cold‐water‐extracted fraction
DMT2: diabetes mellitus type 2
DP: degree of polymerization
FC: fibrillated cellulose
GF: gluten free
GI: glycemic index
HDL: high‐density lipoprotein
HPH: high‐pressure homogenization
HWE/HWF: hot‐water‐extracted fraction
HX: heteroxylan
IAE: intense agitation extract
LDL: low‐density lipoprotein
MD: mean dose
SCFA: short‐chain fatty acid
WAC: water absorption capacity
WSF: whole seed flour

## Author Contributions

**Lucija Strkalj**: conceptualization, writing – original draft, writing – review and editing, visualization. **Gleb E. Yakubov**: conceptualization, writing – review and editing, supervision. **Rachel A. Burton**: conceptualization, writing – review and editing, supervision. **James M. Cowley**: conceptualization, writing – review and editing, visualization, supervision.

## Conflicts of Interest

The authors declare no conflicts of interest.

## Note

1 It is important to note that in the literature, the terms “psyllium,” “psyllium husk,” and “*P. ovata* husk” are used interchangeably, along with traditional names like ispaghula and isabgol, and the expressions that referenced authors originally used are kept in the review.

## References

[crf370297-bib-0001] Addoun, N. , Z. Boual , C. Delattre , et al. 2020. “Structural Features and Rheological Behavior of a Water‐Soluble Polysaccharide Extracted From the Seeds of *Plantago ciliata* Desf.” International Journal of Biological Macromolecules 155: 1333–1341. 10.1016/j.ijbiomac.2019.11.106. 31733242

[crf370297-bib-0002] Addoun, N. , Z. Boual , C. Delattre , et al. 2021. “Beneficial Health Potential of Algerian Polysaccharides Extracted From *Plantago ciliata* Desf. (Septentrional Sahara) Leaves and Seeds.” Applied Sciences 11, no. 9: 4299. 10.3390/app11094299.

[crf370297-bib-0003] Alhasani, A. , M. Anodiyil , M. Corsetti , et al. 2023. “P289 Modelling Psyllium's Inhibitory Effect on Gas Production After FODMAP (Inulin) by Using Divided Doses: A 24‐Hour, Randomised, Placebo‐Controlled Trial.” Gut 72, no. 2: A202–A203. 10.1136/gutjnl-2023-BSG.355.

[crf370297-bib-0004] Alhasani, A. T. , A. A. Modasia , M. Anodiyil , et al. 2025. “Mode of Action of Psyllium in Reducing Gas Production From Inulin and Its Interaction With Colonic Microbiota: A 24‐Hour, Randomized, Placebo‐Controlled Trial in Healthy Human Volunteers.” The Journal of Nutrition 155, no. 3: 839–848. 39732438 10.1016/j.tjnut.2024.12.017 PMC11934246

[crf370297-bib-0005] An, D. , W. Chen , H. Liang , J. Li , P. Zhou , and B. Li . 2026. “Ethanol‐Induced Opposite Viscosity Trends in Psyllium Seed Husk Polysaccharides: The Critical Role of Rhamnogalacturonan‐I in Aggregation Behavior.” Food Hydrocolloids 170: 111717. 10.1016/j.foodhyd.2025.111717.

[crf370297-bib-0006] Anderson, E. , and M. Fireman . 1935. “The Mucilage From Psyllium Seed, *Plantago psyllium*, L.” Journal of Biological Chemistry 109, no. 1: 437–442. 10.1016/S0021-9258(18)75250-2.

[crf370297-bib-0007] Behbahani, B. A. , F. T. Yazdi , F. Shahidi , M. A. Hesarinejad , S. A. Mortazavi , and M. Mohebbi . 2017. “ *Plantago major* Seed Mucilage: Optimization of Extraction and Some Physicochemical and Rheological Aspects.” Carbohydrate Polymers 155: 68–77. 10.1016/j.carbpol.2016.08.051. 27702546

[crf370297-bib-0008] Belorio, M. , and M. Gómez . 2022. “Psyllium: A Useful Functional Ingredient in Food Systems.” Critical Reviews in Food Science and Nutrition 62, no. 2: 527–538. 10.1080/10408398.2020.1822276. 32951436

[crf370297-bib-0009] Benaoun, F. , C. Delattre , Z. Boual , et al. 2017. “Structural Characterization and Rheological Behavior of a Heteroxylan Extracted From *Plantago notata* Lagasca (Plantaginaceae) Seeds.” Carbohydrate Polymers 175: 96–104. 10.1016/j.carbpol.2017.07.056. 28917930

[crf370297-bib-0010] Benson, T. , F. Lavelle , A. McCloat , et al. 2019. “Are the Claims to Blame? A Qualitative Study to Understand the Effects of Nutrition and Health Claims on Perceptions and Consumption of Food.” Nutrients 11, no. 9: 2058. 10.3390/nu11092058. 31480787 PMC6769963

[crf370297-bib-0011] Bhat, S. , A. Vikas , D. Manan , and T. Amin . 2018. “Physicochemical and Textural Properties of Yogurt Fortified With Psyllium (*Plantago ovate*) Husk'.” Journal of Food Processing and Preservation 42, no. 2: e13425. 10.1111/jfpp.13425.

[crf370297-bib-0012] Bilgic, H. , and I. Sensoy . 2023. “Effect of Psyllium and Cellulose Fiber Addition on the Structure and the Starch Digestibility of Bread and Crackers.” Food Structure 35: 100302. 10.1016/j.foostr.2022.100302.

[crf370297-bib-0013] Bliss, D. Z. , H.‐J. Jung , K. Savik , et al. 2001. “Supplementation With Dietary Fiber Improves Fecal Incontinence.” Nursing Research 50, no. 4: 203–213. 11480529 10.1097/00006199-200107000-00004

[crf370297-bib-0014] Bliss, D. Z. , K. Savik , H.‐J. Jung , R. Whitebird , A. Lowry , and X. Sheng . 2014. “Dietary Fiber Supplementation for Fecal Incontinence: A Randomized Clinical Trial.” Research in Nursing & Health 37, no. 5: 367–378. 25155992 10.1002/nur.21616 PMC4296893

[crf370297-bib-0015] Cai, J.‐S. , and J.‐H. Chen . 2014. “The Mechanism of Enterohepatic Circulation in the Formation of Gallstone Disease.” The Journal of Membrane Biology 247, no. 11: 1067–1082. 10.1007/s00232-014-9715-3. 25107305 PMC4207937

[crf370297-bib-0016] Campbell, J. M. , and G. C. Fahey . 1997. “Psyllium and Methylcellulose Fermentation Properties in Relation to Insoluble and Soluble Fiber Standards.” Nutrition Research 17, no. 4: 619–629. 10.1016/S0271-5317(97)00034-1.

[crf370297-bib-0017] Chen, A. , N. Kayrala , M. Trapeau , M. Aoun , and N. Bordenave . 2022. “The Clean Label Trend: An Ineffective Heuristic That Disserves both Consumers and the Food Industry?'.” Comprehensive Reviews in Food Science and Food Safety 21, no. 6: 4921–4938. 10.1111/1541-4337.13031. 36076364

[crf370297-bib-0018] Chen, C. , C. Shang , L. Xin , et al. 2022. “Beneficial Effects of Psyllium on the Prevention and Treatment of Cardiometabolic Diseases.” Food & Function 13, no. 14: 7473–7486. 10.1039/D2FO00560C. 35781477

[crf370297-bib-0019] Cheng, Z. , J. Blackford , Q. Wang , and L. L. Yu . 2009. “Acid Treatment to Improve Psyllium Functionality.” Journal of Functional Foods 1, no. 1: 44–49. 10.1016/j.jff.2008.09.007.

[crf370297-bib-0020] Cicero, A. F. G. , G. Derosa , M. Bove , F. Imola , C. Borghi , and A. V. Gaddi . 2010. “Psyllium Improves Dyslipidaemia, Hyperglycaemia and Hypertension, While Guar Gum Reduces Body Weight More Rapidly in Patients Affected by Metabolic Syndrome Following an AHA Step 2 Diet.” Mediterranean Journal of Nutrition and Metabolism 3, no. 1: 47–54. 10.1007/s12349-009-0056-1.

[crf370297-bib-0021] Cikrikci Erunsal, S. , M. B. Karabiyik , K. S. Kirdi , and H. N. Inac . 2023. “Development of Psyllium Seed Husk‐Based Colorimetric Indicator by Different Homogenization Methods.” Chemical Papers 77, no. 3: 1729–1740. 10.1007/s11696-023-02677-8.

[crf370297-bib-0022] Cowley, J. M. , and R. A. Burton . 2021. “The Goo‐d Stuff: *Plantago* as a Myxospermous Model With Modern Utility.” New Phytologist 229, no. 4: 1917–1923. 10.1111/nph.17095. 33220085

[crf370297-bib-0023] Cowley, J. M. , L. Herliana , K. A. Neumann , S. Ciani , V. Cerne , and R. A. Burton . 2020. “A Small‐Scale Fractionation Pipeline for Rapid Analysis of Seed Mucilage Characteristics.” Plant Methods 16, no. 1: 20. 10.1186/s13007-020-00569-6. 32123537 PMC7038624

[crf370297-bib-0024] Cowley, J. M. , D. L. McNeil , K. Y. Lui , et al. 2022. “Rain Events at Maturity Severely Impact the Seed Quality of Psyllium (*Plantago ovata* Forssk.).” Journal of Agronomy and Crop Science 208, no. 4: 567–581. 10.1111/jac.12603.

[crf370297-bib-0025] Cowley, J. M. , L. A. O'Donovan , and R. A. Burton . 2021. “The Composition of Australian *Plantago* Seeds Highlights Their Potential as Nutritionally‐Rich Functional Food Ingredients.” Scientific Reports 11, no. 1: 12692. 10.1038/s41598-021-92114-1. 34135417 PMC8209032

[crf370297-bib-0026] Cowley, J. M. , Y. Ren , L. Štrkalj , T. J. Foster , and R. A. Burton . 2025. “The Influence of Mucilage‐Rich Flours From Diverse Australian *Plantago* Species on the Pasting, Storage, and Gluten‐Free Breadmaking Properties of Rice Flour.” Food Hydrocolloids 160, no. Pt. 1: 110788. 10.1016/j.foodhyd.2024.110788.

[crf370297-bib-0150] Davidson, M. , L. Dugan , J. Burns , D. Sugimoto , K. Story , and K. Drennan . 1996. “A Psyllium‐enriched Cereal for the Treatment of Hypercholesterolemia in Children: a Controlled, Double‐blind, Crossover Study.” The American Journal of Clinical Nutrition 63, no. 1: 96–102. 10.1093/ajcn/63.1.96. 8604676

[crf370297-bib-0149] Davidson, M. , K. Maki , J. Kong , et al. 1998. “Long‐term Effects of Consuming Foods Containing Psyllium Seed Husk on Serum Lipids in Subjects With Hypercholesterolemia.” The American Journal of Clinical Nutrition 67, no. 3: 367–376. 10.1093/ajcn/67.3.367. 9497178

[crf370297-bib-0027] Dhar, M. K. , S. Kaul , S. Sareen , and A. K. Koul . 2005. “ *Plantago ovata*: Genetic Diversity, Cultivation, Utilization and Chemistry.” Plant Genetic Resources 3, no. 2: 252–263. 10.1079/PGR200582.

[crf370297-bib-0028] Dikeman, C. L. , M. R. Murphy , and G. C. Fahey . 2006. “Dietary Fibers Affect Viscosity of Solutions and Simulated Human Gastric and Small Intestinal Digesta.” The Journal of Nutrition 136, no. 4: 913–919. 10.1093/jn/136.4.913. 16549450

[crf370297-bib-0029] Edwards, S. , M. F. Chaplin , A. D. Blackwood , and P. W. Dettmar . 2003. “Primary Structure of Arabinoxylans of Ispaghula Husk and Wheat Bran.” Proceedings of the Nutrition Society 62, no. 1: 217–222. 10.1079/PNS2003202. 12756970

[crf370297-bib-0030] European Food Safety Authority (EFSA) . 2010. “Scientific Opinion on the Substantiation of Health Claims Related to Dietary Fibre (ID 744, 745, 746, 748, 749, 753, 803, 810, 855, 1415, 1416, 4308, 4330) pursuant to Article 13(1) of Regulation (EC) No 1924/2006.” EFSA Journal 8, no. 10: 1735. 10.2903/j.efsa.2010.1735.

[crf370297-bib-0031] European Parliament . 2006. “Regulation (EC) No 1924/2006 of the European Parliament and of the Council on Nutrition and Health Made on Foods.” https://theitalian.ca/wp‐content/uploads/2022/12/Rules‐on‐nutrition‐claims_EN_TXT‐Regles‐sur‐les‐allegations‐nutritionnelles‐FR_TXT.pdf.

[crf370297-bib-0032] Farahnaky, A. , H. Askari , M. Majzoobi , and G. Mesbahi . 2010. “The Impact of Concentration, Temperature and pH on Dynamic Rheology of Psyllium Gels.” Journal of Food Engineering 100, no. 2: 294–301. 10.1016/j.jfoodeng.2010.04.012.

[crf370297-bib-0033] Ferjančič, B. , S. Kugler , M. Korošec , T. Polak , and J. Bertoncelj . 2021. “Development of Low‐Fat Chicken Bologna Sausages Enriched With Inulin, Oat Fibre or Psyllium.” International Journal of Food Science & Technology 56, no. 4: 1818–1828. 10.1111/ijfs.14808.

[crf370297-bib-0034] Figueroa, L. E. , and D. B. Genovese . 2018. “Pectin Gels Enriched With Dietary Fibre for the Development of Healthy Confectionery Jams.” Food Technology and Biotechnology 56, no. 3: 441–453. 10.17113/ftb.56.03.18.5641. 30510487 PMC6233018

[crf370297-bib-0035] Figueroa, L. E. , and D. B. Genovese . 2019. “Fruit Jellies Enriched With Dietary Fibre: Development and Characterization of a Novel Functional Food Product.” LWT ‐ Food Science and Technology 111: 423–428. 10.1016/j.lwt.2019.05.031.

[crf370297-bib-0036] Fischer, M. H. , N. Yu , G. R. Gray , J. Ralph , L. Anderson , and J. A. Marlett . 2004. “The Gel‐Forming Polysaccharide of Psyllium Husk (*Plantago ovata* Forsk).” Carbohydrate Research 339, no. 11: 2009–2017. 10.1016/j.carres.2004.05.023. 15261594

[crf370297-bib-0037] Food and Drug Administration . 2002. “Food Labeling: Health Claims; Soluble Dietary Fiber From Certain Foods and Coronary Heart Disease.” https://www.govinfo.gov/content/pkg/FR‐2002‐10‐02/pdf/02‐25067.pdf.

[crf370297-bib-0038] Ford, A. C. , P. Moayyedi , W. D. Chey , et al. 2018. “American College of Gastroenterology Monograph on Management of Irritable Bowel Syndrome.” American Journal of Gastroenterology 113: 1–18. 10.1038/s41395-018-0084-x. 29950604

[crf370297-bib-0039] Foschia, M. , D. Peressini , A. Sensidoni , and C. Stephen Brennan . 2013. “The Effects of Dietary Fibre Addition on the Quality of Common Cereal Products.” Journal of Cereal Science 58, no. 2: 216–227. 10.1016/j.jcs.2013.05.010.

[crf370297-bib-0040] Fradinho, P. , M. C. Nunes , and A. Raymundo . 2015. “Developing Consumer Acceptable Biscuits Enriched With Psyllium Fibre.” Journal of Food Science and Technology 52, no. 8: 4830–4840. 10.1007/s13197-014-1549-6. 26243903 PMC4519454

[crf370297-bib-0041] Fradinho, P. , R. Soares , A. Niccolai , I. Sousa , and A. Raymundo . 2020. “Psyllium Husk Gel to Reinforce Structure of Gluten‐Free Pasta?'.” LWT ‐ Food Science and Technology 131: 109787. 10.1016/j.lwt.2020.109787.

[crf370297-bib-0042] Franco, E. A. N. , A. Sanches‐Silva , R. Ribeiro‐Santos , and N. R. De Melo . 2020. “Psyllium (*Plantago ovata* Forsk): From Evidence of Health Benefits to Its Food Application.” Trends in Food Science & Technology 96: 166–175. 10.1016/j.tifs.2019.12.006.

[crf370297-bib-0043] Fratelli, C. , F. Garcia Santos , D. G. Muniz , S. Habu , A. R. C. Braga , and V. D. Capriles . 2021. “Psyllium Improves the Quality and Shelf Life of Gluten‐Free Bread.” Foods 10, no. 5: 954. 10.3390/foods10050954. 33925416 PMC8145964

[crf370297-bib-0044] Fratelli, C. , D. G. Muniz , F. G. Santos , and V. D. Capriles . 2018. “Modelling the Effects of Psyllium and Water in Gluten‐Free Bread: An Approach to Improve the Bread Quality and Glycemic Response.” Journal of Functional Foods 42: 339–345. 10.1016/j.jff.2018.01.015.

[crf370297-bib-0045] Fukudo, S. , T. Okumura , M. Inamori , et al. 2021. “Evidence‐Based Clinical Practice Guidelines for Irritable Bowel Syndrome 2020.” Journal of Gastroenterology 56, no. 3: 193–217. 10.1007/s00535-020-01746-z. 33538894 PMC7932982

[crf370297-bib-1145] Garg, P. 2017. “Physiologic Management of Chronic Constipation: Let's Feed it.” Digestive Diseases and Sciences 62, no. 11: 3254–3255. 28948409 10.1007/s10620-017-4769-6

[crf370297-bib-0146] Gholami, Z. , and Z. Paknahad . 2024. “The Effect of Psyllium Consumption on Blood Pressure: Systematic Review and Dose‐response Meta‐analysis of Randomized Controlled Trials.” Food Science & Nutrition 12, no. 10: 7075–7087. Portico. 10.1002/fsn3.3863. 39479650 PMC11521634

[crf370297-bib-0046] Giacco, R. , G. Costabile , and G. Riccardi . 2016. “Metabolic Effects of Dietary Carbohydrates: The Importance of Food Digestion.” Food Research International 88: 336–341. 10.1016/j.foodres.2015.10.026.

[crf370297-bib-0047] Gibb, R. D. , J. W. McRorie , D. A. Russell , V. Hasselblad , and D. A. D'Alessio . 2015. “Psyllium Fiber Improves Glycemic Control Proportional to Loss of Glycemic Control: A Meta‐Analysis of Data in Euglycemic Subjects, Patients at Risk of Type 2 Diabetes Mellitus, and Patients Being Treated for Type 2 Diabetes Mellitus.” The American Journal of Clinical Nutrition 102, no. 6: 1604–1614. 10.3945/ajcn.115.106989. 26561625

[crf370297-bib-0048] Gibb, R. D. , K. J. Sloan , and J. W. McRorie . 2023. “Psyllium Is a Natural Nonfermented Gel‐Forming Fiber That Is Effective for Weight Loss: A Comprehensive Review and Meta‐Analysis.” Journal of the American Association of Nurse Practitioners 35, no. 8: 468–476. 10.1097/JXX.0000000000000882. 37163454 PMC10389520

[crf370297-bib-0049] Gidley, M. J. , and G. E. Yakubov . 2019. “Functional Categorisation of Dietary Fibre in Foods: Beyond “Soluble” vs “Insoluble”.” Trends in Food Science & Technology 86: 563–568. 10.1016/j.tifs.2018.12.006.

[crf370297-bib-0050] Goldsborough, E. , N. Osuji , and M. J. Blaha . 2022. “Assessment of Cardiovascular Disease Risk.” Endocrinology and Metabolism Clinics of North America 51, no. 3: 483–509. 10.1016/j.ecl.2022.02.005. 35963625

[crf370297-bib-0051] Govt India Dept. of Commerce . 2024. “Psyllium Seed (Isobgul) 12119013 Export: Commodity‐Wise.” https://tradestat.commerce.gov.in/eidb/Default.asp.

[crf370297-bib-0052] Gunn, D. , Z. Abbas , H. C. Harris , et al. 2022. “Psyllium Reduces Inulin‐Induced Colonic Gas Production in IBS: MRI and In Vitro Fermentation Studies.” Gut 71, no. 5: 919–927. 10.1136/gutjnl-2021-324784. 34353864 PMC8995815

[crf370297-bib-0053] Guo, Q. , S. W. Cui , Q. Wang , and J. Christopher Young . 2008. “Fractionation and Physicochemical Characterization of Psyllium Gum.” Carbohydrate Polymers 73, no. 1: 35–43. 10.1016/j.carbpol.2007.11.001.

[crf370297-bib-0054] Haque, A. , R. K. Richardson , E. R. Morris , and I. C. M. Dea . 1993. “Xanthan‐Like “Weak Gel” Rheology From Dispersions of Ispaghula Seed Husk'.” Carbohydrate Polymers 22, no. 4: 223–232. 10.1016/0144-8617(93)90124-M.

[crf370297-bib-0055] Harris, H. C. , N. Pereira , T. Koev , Y. Z. Khimyak , G. E. Yakubov , and F. J. Warren . 2023. “The Impact of Psyllium Gelation Behaviour on In Vitro Colonic Fermentation Properties.” Food Hydrocolloids 139: 108543. 10.1016/j.foodhyd.2023.108543.

[crf370297-bib-0151] Hefny, A. F. , A. Z. Ayad , N. Matev , and M. O. Bashir . 2018. “Intestinal Obstruction Caused by a Laxative Drug (Psyllium): a Case Report and Review of the Literature.” International Journal of Surgery Case Reports 52: 59–62. 10.1016/j.ijscr.2018.10.001. 30321826 PMC6197948

[crf370297-bib-0056] Herliana, L. , J. M. Cowley , L. A. O'Donovan , T. Bianco‐Miotto , and R. A. Burton . 2025. “Morphological and Developmental Analysis of *Plantago* Spp. Seed Capsules Reveals Key Features of the Dehiscence Zones.” Annals of Botany 135, no. 7: mcaf044. 10.1093/aob/mcaf044. PMC12358031 40084499

[crf370297-bib-0057] Herliana, L. , J. G. Schwerdt , T. R. Neumann , et al. 2023. “A Chromosome‐Level Genome Assembly of *Plantago ovata* .” Scientific Reports 13, no. 1: 1528. 10.1038/s41598-022-25078-5. 36707685 PMC9883528

[crf370297-bib-0058] Hesarinejad, M. A. , M. S. Jokandan , M. A. Mohammadifar , et al. 2018. “The Effects of Concentration and Heating‐Cooling Rate on Rheological Properties of *Plantago lanceolata* Seed Mucilage.” International Journal of Biological Macromolecules 115: 1260–1266. 10.1016/j.ijbiomac.2017.10.102. 29054524

[crf370297-bib-0059] Hirst, E. L. 1951. “Studies on Seed Mucilages. Part VI. The Seed Mucilage of *Plantago arenaria* .” Journal of the Chemical Society (Resumed) 189–198.

[crf370297-bib-0060] Hu, J.‐L. , S.‐P. Nie , C. Li , Z.‐H. Fu , and M.‐Y. Xie . 2013. “Microbial Short‐Chain Fatty Acid Production and Extracellular Enzymes Activities During In Vitro Fermentation of Polysaccharides From the Seeds of *Plantago asiatica* L. Treated With Microwave Irradiation.” Journal of Agricultural and Food Chemistry 61, no. 25: 6092–6101. 10.1021/jf401877j. 23738978

[crf370297-bib-0061] Hu, J.‐L. , S.‐P. Nie , and M.‐Y. Xie . 2013. “High Pressure Homogenization Increases Antioxidant Capacity and Short‐Chain Fatty Acid Yield of Polysaccharide From Seeds of *Plantago asiatica* L.” Food Chemistry 138, no. 4: 2338–2345. 10.1016/j.foodchem.2012.12.016. 23497894

[crf370297-bib-0062] Huang, D. , Q. Xia , F. Li , W. Yang , S. Nie , and M. Xie . 2018. “Attenuation of Intestinal Inflammation of Polysaccharides From the Seeds of *Plantago asiatica* L. as Affected by Ultrasonication.” Journal of Food Biochemistry 42, no. 6: e12656. 10.1111/jfbc.12656.

[crf370297-bib-0063] Jalanka, J. , G. Major , K. Murray , et al. 2019. “The Effect of Psyllium Husk on Intestinal Microbiota in Constipated Patients and Healthy Controls.” International Journal of Molecular Sciences 20, no. 2: 433. 10.3390/ijms20020433. 30669509 PMC6358997

[crf370297-bib-0064] Jensen, J. K. , N. Johnson , and C. G. Wilkerson . 2013. “Discovery of Diversity in Xylan Biosynthetic Genes by Transcriptional Profiling of a Heteroxylan Containing Mucilaginous Tissue.” Frontiers in Plant Science 4: 183. 10.3389/fpls.2013.00183. 23761806 PMC3675317

[crf370297-bib-0065] Jensen, J. K. , N. R. Johnson , and C. G. Wilkerson . 2014. “ *Arabidopsis thaliana* irx 10 and Two Related Proteins From Psyllium and * physcomitrella patens* Are Xylan Xylosyltransferases.” The Plant Journal 80, no. 2: 207–215. 10.1111/tpj.12641. 25139408

[crf370297-bib-0066] Kaczmarczyk, K. , J. Kruk , P. Ptaszek , and A. Ptaszek . 2023. “ *Plantago ovata* Husk: An Investigation of Raw Aqueous Extracts. Osmotic, Hydrodynamic and Complex Rheological Characterisation.” Molecules 28, no. 4: 1660. 10.3390/molecules28041660. 36838648 PMC9962041

[crf370297-bib-0067] Kajzer, M. , and A. Diowksz . 2021. “The Clean Label Concept: Novel Approaches in Gluten‐Free Breadmaking.” Applied Sciences 11, no. 13: 6129. 10.3390/app11136129.

[crf370297-bib-0068] Kennedy, J. F. , J. S. Sandhu , and D. A. T. Southgate . 1979. “Structural Data for the Carbohydrate of Ispaghula Husk Ex *Plantago ovata* Forsk.” Carbohydrate Research 75: 265–274.

[crf370297-bib-0069] Kiszonas, A. M. , E. P. Fuerst , and C. F. Morris . 2013. “Wheat Arabinoxylan Structure Provides Insight Into Function.” Cereal Chemistry 90, no. 4: 387–395. 10.1094/CCHEM-02-13-0025-FI.

[crf370297-bib-0070] Kour, B. , S. Kotwal , M. K. Dhar , and S. Kaul . 2016. “Genetic Diversity Analysis in *Plantago ovata* and Some of Its Wild Allies Using RAPD Markers.” Russian Agricultural Sciences 42, no. 1: 37–41. 10.3103/S1068367416010055.

[crf370297-bib-0071] Kristensen, M. , and M. G. Jensen . 2011. “Dietary Fibres in the Regulation of Appetite and Food Intake. Importance of Viscosity.” Appetite 56, no. 1: 65–70. 10.1016/j.appet.2010.11.147. 21115081

[crf370297-bib-0072] Laidlaw, A. , and E. G. V. Percival . 1949. “Studies on Seed Mucilages. Part III. Examination of a Polysaccharide Extracted From the Seeds of *Plantago ovata* Forsk.” Journal of the Chemical Society (Resumed) 1600–1607.

[crf370297-bib-0073] Levengood, H. , Y. Dou , J. Fan , et al. 2023. “Agrobacterium Tumefaciens‐Mediated Genetic Transformation of Narrowleaf Plantain.” Journal of Visualized Experiments 193: 64777. 10.3791/64777. 37010286

[crf370297-bib-0074] Liu, W. , Z. Xie , B. Zhang , et al. 2010. “Effects of Hydroxypropylation on the Functional Properties of Psyllium.” Journal of Agricultural and Food Chemistry 58, no. 3: 1615–1621. 10.1021/jf903691z. 20085281

[crf370297-bib-0075] Liu, W. , B. Zhang , Q. Wang , et al. 2010. “Effects of Sulfation on the Physicochemical and Functional Properties of Psyllium.” Journal of Agricultural and Food Chemistry 58, no. 1: 172–179. 10.1021/jf902731p. 20000369

[crf370297-bib-0076] Lovegrove, A. , M. D. Wilkinson , J. Freeman , et al. 2013. “RNA Interference Suppression of Genes in Glycosyl Transferase Families 43 and 47 in Wheat Starchy Endosperm Causes Large Decreases in Arabinoxylan Content.” Plant Physiology 163, no. 1: 95–107. 10.1104/pp.113.222653. 23878080 PMC3762668

[crf370297-bib-0077] Lyu, S. , Q. Mei , H. Liu , et al. 2023. “Genome Assembly of the Pioneer Species *Plantago major* L. (Plantaginaceae) Provides Insight Into Its Global Distribution and Adaptation to Metal‐Contaminated Soil'.” DNA Research 30, no. 4: dsad013. 10.1093/dnares/dsad013. 37228100 PMC10254747

[crf370297-bib-0078] Major, G. , K. Murray , G. Singh , et al. 2018. “Demonstration of Differences in Colonic Volumes, Transit, Chyme Consistency, and Response to Psyllium Between Healthy and Constipated Subjects Using Magnetic Resonance Imaging.” Neurogastroenterology & Motility 30, no. 9: e13400. 10.1111/nmo.13400. 30062794

[crf370297-bib-0079] Maljaars, P. W. J. , H. P. F. Peters , D. J. Mela , and A. A. M. Masclee . 2008. “Ileal Brake: A Sensible Food Target for Appetite Control. A Review.” Physiology & Behavior 95, no. 3: 271–281. 10.1016/j.physbeh.2008.07.018. 18692080

[crf370297-bib-0080] Mallikarjunan, N. , R. Deshpande , and S. N. Jamdar . 2021. “Radiation Processing of Psyllium and Its Application in Development of Low Glycaemic Food.” Radiation Physics and Chemistry 186: 109477. 10.1016/j.radphyschem.2021.109477.

[crf370297-bib-0081] Mallikarjunan, N. , D. Rajalakshmi , D. K. Maurya , and S. N. Jamdar . 2024. “Modifying Rheological Properties of Psyllium by Gamma Irradiation Enables Development of Low Glycaemic Index Food With a Predicted Gastrointestinal Tolerance.” International Journal of Biological Macromolecules 257: 128625. 10.1016/j.ijbiomac.2023.128625. 38065450

[crf370297-bib-0082] Mancebo, C. M. , M. Á. S. Miguel , M. M. Martínez , and M. Gómez . 2015. “Optimisation of Rheological Properties of Gluten‐Free Doughs With HPMC, Psyllium and Different Levels of Water.” Journal of Cereal Science 61: 8–15. 10.1016/j.jcs.2014.10.005.

[crf370297-bib-0083] Mariotti, M. , M. Lucisano , M. A. Pagani , and P. K. W. Ng . 2009. “The Role of Corn Starch, Amaranth Flour, Pea Isolate, and Psyllium Flour on the Rheological Properties and the Ultrastructure of Gluten‐Free Doughs.” Food Research International 42, no. 8: 963–975. 10.1016/j.foodres.2009.04.017.

[crf370297-bib-0084] Marlett, J. A. , and M. H. Fischer . 2003. “The Active Fraction of Psyllium Seed Husk.” Proceedings of the Nutrition Society 62, no. 1: 207–209. 10.1079/PNS2002201. 12749348

[crf370297-bib-0085] Marlett, J. A. , T. M. Kajs , and M. H. Fischer . 2000. “An Unfermented Gel Component of Psyllium Seed Husk Promotes Laxation as a Lubricant in Humans.” The American Journal of Clinical Nutrition 72, no. 3: 784–789. 10.1093/ajcn/72.3.784. 10966900

[crf370297-bib-0086] Marteau, P. , B. Flourie , C. Cherbut , et al. 1994. “Digestibility and Bulking Effect of Ispaghula Husks in Healthy Humans.” Gut 35, no. 12: 1747–1752. 10.1136/gut.35.12.1747. 7829013 PMC1375264

[crf370297-bib-0148] Martellet, M. C. , F. Majolo , R. G. Ducati , C. F. Volken de Souza , and M. I. Goettert . 2022. “Probiotic Applications Associated With Psyllium fiber as Prebiotics Geared to a Healthy Intestinal Microbiota: a Review.” Nutrition (Burbank, Los Angeles County, California) 103–104. 10.1016/j.nut.2022.111772. 35930916

[crf370297-bib-0087] McGee, R. , G. H. Dean , D. Wu , Y. Zhang , S. D. Mansfield , and G. W. Haughn . 2021. “Pectin Modification in Seed Coat Mucilage by *In Vivo* Expression of Rhamnogalacturonan‐I‐ and Homogalacturonan‐Degrading Enzymes.” Plant and Cell Physiology 62, no. 12: 1912–1926. 10.1093/pcp/pcab077. 34059917

[crf370297-bib-0088] McKeown, N. M. , G. C. Fahey , J. Slavin , and J.‐W. Van Der Kamp . 2022. “Fibre Intake for Optimal Health: How Can Healthcare Professionals Support People to Reach Dietary Recommendations?'.” BMJ 378: e054370. 10.1136/bmj-2020-054370. 35858693 PMC9298262

[crf370297-bib-0089] McRorie, J. W. , R. D. Gibb , K. J. Sloan , and N. M. McKeown . 2021. “Psyllium: The Gel‐Forming Nonfermented Isolated Fiber That Delivers Multiple Fiber‐Related Health Benefits'.” Nutrition Today 56, no. 4: 169–182. 10.1097/NT.0000000000000489.

[crf370297-bib-0090] McRorie, J. W. , R. D. Gibb , J. B. Womack , and D. J. Pambianco . 2017. “Psyllium Is Superior to Wheat Dextrin for Lowering Elevated Serum Cholesterol.” Nutrition Today 52, no. 6: 289–294. 10.1097/NT.0000000000000243.

[crf370297-bib-0091] McRorie, J. W. , and N. M. McKeown . 2017. “Understanding the Physics of Functional Fibers in the Gastrointestinal Tract: An Evidence‐Based Approach to Resolving Enduring Misconceptions About Insoluble and Soluble Fiber.” Journal of the Academy of Nutrition and Dietetics 117, no. 2: 251–264. 10.1016/j.jand.2016.09.021. 27863994

[crf370297-bib-0092] Meldrum, O. W. , and G. E. Yakubov . 2025. “Journey of Dietary Fiber Along the Gastrointestinal Tract: Role of Physical Interactions, Mucus, and Biochemical Transformations.” Critical Reviews in Food Science and Nutrition 65, no. 22: 4264–4292. 10.1080/10408398.2024.2390556. 39141568

[crf370297-bib-0093] Mironeasa, S. , and G. G. Codină . 2023. “Optimization of Bread Quality of Wheat Flour With Psyllium Addition by Using Response Surface Methodology.” Journal of Culinary Science & Technology 21, no. 3: 371–386. 10.1080/15428052.2021.1948480.

[crf370297-bib-0094] Moayyedi, P. , C. N. Andrews , G. MacQueen , et al. 2019. “Canadian Association of Gastroenterology Clinical Practice Guideline for the Management of Irritable Bowel Syndrome (IBS).” Journal of the Canadian Association of Gastroenterology 2, no. 1: 6–29. 10.1093/jcag/gwy071. 31294724 PMC6507291

[crf370297-bib-0095] Mullan, J. , and E. G. V. Percival . 1940. “280. Studies on Seed Mucilage. Part I. A Preliminary Examination of the Mucilaginous Polysaccharide From the Seeds of *Plantago lanceolata* .” Journal of the Chemical Society (Resumed) 1501–1506.

[crf370297-bib-0096] Nadkarni, K. M. 1927. Indian Materia Medica. 2nd ed. Ramdas Bhatkal for Bombay Popular Prakashan.

[crf370297-bib-0097] Nagel, A. , S. Sirisakulwat , R. Carle , and S. Neidhart . 2014. “An Acetate–Hydroxide Gradient for the Quantitation of the Neutral Sugar and Uronic Acid Profile of Pectins by HPAEC‐PAD Without Postcolumn pH Adjustment.” Journal of Agricultural and Food Chemistry 62, no. 9: 2037–2048. 10.1021/jf404626d. 24547908

[crf370297-bib-0098] Niknam, R. , B. Ghanbarzadeh , A. Ayaseh , and F. Rezagholi . 2018. “The Effects of *Plantago major* Seed Gum on Steady and Dynamic Oscillatory Shear Rheology of Sunflower Oil‐in‐Water Emulsions.” Journal of Texture Studies 49, no. 5: 536–547. 10.1111/jtxs.12352. 29975418

[crf370297-bib-0099] Niknam, R. , B. Ghanbarzadeh , A. Ayaseh , and F. Rezagholi . 2020. “The Hydrocolloid Extracted From *Plantago major* Seed: Effects on Emulsifying and Foaming Properties.” Journal of Dispersion Science and Technology 41, no. 5: 667–673. 10.1080/01932691.2019.1610426.

[crf370297-bib-0100] Niu, Y. , Q. Xia , W. Jung , and L. Yu . 2019. “Polysaccharides‐Protein Interaction of Psyllium and Whey Protein With Their Texture and Bile Acid Binding Activity.” International Journal of Biological Macromolecules 126: 215–220. 10.1016/j.ijbiomac.2018.12.221. 30590142

[crf370297-bib-0101] Niu, Y. , Z. Xie , J. J. Hao , W. B. Yao , J. Yue , and L. L. Yu . 2012. “Preparation of Succinylated Derivatives of Psyllium and Their Physicochemical and Bile Acid‐Binding Properties.” Food Chemistry 132, no. 2: 1025–1032. 10.1016/j.foodchem.2011.11.090.

[crf370297-bib-0102] Niu, Y. , Z. Xie , H. Zhang , Y. Sheng , and L. L. Yu . 2013. “Effects of Structural Modifications on Physicochemical and Bile Acid‐Binding Properties of Psyllium.” Journal of Agricultural and Food Chemistry 61, no. 3: 596–601. 10.1021/jf3043117. 23286525

[crf370297-bib-0103] Noguerol, A. T. , M. M. Igual , and M. J. Pagán . 2022. “Developing Psyllium Fibre Gel‐Based Foods: Physicochemical, Nutritional, Optical and Mechanical Properties.” Food Hydrocolloids 122: 107108. 10.1016/j.foodhyd.2021.107108.

[crf370297-bib-0104] Noguerol, A. T. , V. Larrea , and M. Jesús Pagán . 2022. “The Effect of Psyllium (*Plantago ovata* Forsk) Fibres on the Mechanical and Physicochemical Characteristics of Plant‐Based Sausages.” European Food Research and Technology 248, no. 10: 2483–2496. 10.1007/s00217-022-04063-2. 35818621 PMC9261230

[crf370297-bib-0105] Pal, S. , and S. Radavelli‐Bagatini . 2012. “Effects of Psyllium on Metabolic Syndrome Risk Factors.” Obesity Reviews 13, no. 11: 1034–1047. 10.1111/j.1467-789X.2012.01020.x. 22863407

[crf370297-bib-0106] Pan, V. S. , C. Girvin , and E. F. LoPresti . 2022. “Anchorage by Seed Mucilage Prevents Seed Dislodgement in High Surface Flow: A Mechanistic Investigation.” Annals of Botany 129, no. 7: 817–830. 10.1093/aob/mcac045. 35325924 PMC9292590

[crf370297-bib-0107] Pavlovich‐Abril, A. , O. Rouzaud‐Sández , P. Torres , and R. Maribel Robles‐Sánchez . 2012. “Cereal Bran and Wholegrain as a Source of Dietary Fibre: Technological and Health Aspects'.” International Journal of Food Sciences and Nutrition 63, no. 7: 882–892. 10.3109/09637486.2012.676030. 22486424

[crf370297-bib-0108] Pejcz, E. , R. Spychaj , A. Wojciechowicz‐Budzisz , and Z. Gil . 2018. “The Effect of *Plantago* Seeds and Husk on Wheat Dough and Bread Functional Properties.” LWT ‐ Food Science and Technology 96: 371–377. 10.1016/j.lwt.2018.05.060.

[crf370297-bib-0109] Phan, J. L. , J. M. Cowley , K. A. Neumann , L. Herliana , L. A. O'Donovan , and R. A. Burton . 2020. “The Novel Features of *Plantago ovata* Seed Mucilage Accumulation, Storage and Release.” Scientific Reports 10: 11766. 32678191 10.1038/s41598-020-68685-w PMC7366641

[crf370297-bib-0110] Phan, J. L. , M. R. Tucker , S. F. Khor , et al. 2016. “Differences in Glycosyltransferase Family 61 Accompany Variation in Seed Coat Mucilage Composition in *Plantago* Spp.” Journal of Experimental Botany 67, no. 22: 6481–6495. 10.1093/jxb/erw424. 27856710 PMC5181589

[crf370297-bib-0111] Pollet, A. , V. Van Craeyveld , T. Van De Wiele , W. Verstraete , J. A. Delcour , and C. M. Courtin . 2012. “In Vitro Fermentation of Arabinoxylan Oligosaccharides and Low Molecular Mass Arabinoxylans With Different Structural Properties From Wheat (*Triticum aestivum* L.) Bran and Psyllium (*Plantago ovata* Forsk) Seed Husk.” Journal of Agricultural and Food Chemistry 60, no. 4: 946–954. 10.1021/jf203820j. 22224418

[crf370297-bib-0112] Raymundo, A. , P. Fradinho , and M. C. Nunes . 2014. “Effect of Psyllium Fibre Content on the Textural and Rheological Characteristics of Biscuit and Biscuit Dough.” Bioactive Carbohydrates and Dietary Fibre 3, no. 2: 96–105. 10.1016/j.bcdf.2014.03.001.

[crf370297-bib-0113] Ren, Y. , B. R. Linter , and T. J. Foster . 2020. “Cellulose Fibrillation and Interaction With Psyllium Seed Husk Heteroxylan.” Food Hydrocolloids 104: 105725. 10.1016/j.foodhyd.2020.105725.

[crf370297-bib-0114] Ren, Y. , B. R. Linter , R. Linforth , and T. J. Foster . 2020. “A Comprehensive Investigation of Gluten Free Bread Dough Rheology, Proving and Baking Performance and Bread Qualities by Response Surface Design and Principal Component Analysis.” Food & Function 11, no. 6: 5333–5345. 10.1039/D0FO00115E. 32459258

[crf370297-bib-0115] Ren, Y. , G. E. Yakubov , B. R. Linter , W. MacNaughtan , and T. J. Foster . 2020. “Temperature Fractionation, Physicochemical and Rheological Analysis of Psyllium Seed Husk Heteroxylan.” Food Hydrocolloids 104: 105737. 10.1016/j.foodhyd.2020.105737.

[crf370297-bib-0116] Ribas, S. A. , D. B. Cunha , R. Sichieri , and L. C. S. Da Silva . 2015. “Effects of Psyllium on LDL‐Cholesterol Concentrations in Brazilian Children and Adolescents: A Randomised, Placebo‐Controlled, Parallel Clinical Trial.” British Journal of Nutrition 113, no. 1: 134–141. 10.1017/S0007114514003419. 25391814

[crf370297-bib-0117] Roman, L. , M. Belorio , and M. Gomez . 2019. “Gluten‐Free Breads: The Gap between Research and Commercial Reality.” Comprehensive Reviews in Food Science and Food Safety 18, no. 3: 690–702. 10.1111/1541-4337.12437. 33336920

[crf370297-bib-0118] Sandhu, J. S. , G. J. Hudson , and J. F. Kennedy . 1981. “The Gel Nature and Structure of the Carbohydrate of Ispaghula Husk Ex *Plantago ovata* Forsk.” Carbohydrate Research 93, no. 2: 247–259. 10.1016/S0008-6215(00)80854-X.

[crf370297-bib-0153] van der Schoot, A. , C. Drysdale , K. Whelan , and E. Dimidi . 2022. “The Effect of Fiber Supplementation on Chronic Constipation in Adults: an Updated Systematic Review and Meta‐Analysis of Randomized Controlled Trials.” The American Journal of Clinical Nutrition 116, no. 4: 953–969. 10.1093/ajcn/nqac184. 35816465 PMC9535527

[crf370297-bib-0119] Sevilmis, B. , and I. Sensoy . 2022. “Effects of Psyllium Fiber on In Vitro Digestion and Structure of Different Types of Starches.” Journal of the Science of Food and Agriculture 102, no. 8: 3213–3226. 10.1002/jsfa.11664. 34796511

[crf370297-bib-0120] Shanahan, C. J. , R. D. Gibb , J. W. McRorie , J. M. Brum , and M. E. Ritchey . 2019. “Economic Impact Analysis of the Coronary Heart Disease‐Attributed Health Care Cost Savings and Productivity Gains From the Use of Psyllium.” Nutrition & Food Science 50, no. 3: 497–513. 10.1108/NFS-03-2019-0067.

[crf370297-bib-0121] Sharma, P. K. , and A. K. Koul . 1986. “Mucilage in Seeds of *Plantago ovata* and Its Wild Allies.” Journal of Ethnopharmacology 17, no. 3: 289–295. 10.1016/0378-8741(86)90118-2. 3807392

[crf370297-bib-0122] Simopoulos, A. P. 2002. “The Importance of the Ratio of Omega‐6/Omega‐3 Essential Fatty Acids.” Biomedicine & Pharmacotherapy 56, no. 8: 365–379. 12442909 10.1016/s0753-3322(02)00253-6

[crf370297-bib-0123] Singh, J. , A. Dartois , and L. Kaur . 2010. “Starch Digestibility in Food Matrix: A Review.” Trends in Food Science & Technology 21, no. 4: 168–180. 10.1016/j.tifs.2009.12.001.

[crf370297-bib-0124] Slavin, J. , and H. Green . 2007. “Dietary Fibre and Satiety.” Nutrition Bulletin 32: no. Suppl I: 32–42.

[crf370297-bib-0125] Tejada‐Ortigoza, V. , L. E. Garcia‐Amezquita , S. O. Serna‐Saldívar , and J. Welti‐Chanes . 2016. “Advances in the Functional Characterization and Extraction Processes of Dietary Fiber.” Food Engineering Reviews 8: 251–271.

[crf370297-bib-0154] Tominaga, Y. , I. Hirayama , M. Nonaka , T. Yano , and M. Ishii . 2023. “Intestinal Obstruction Caused by Consuming Diet Food Containing Psyllium.” Acute Medicine & Surgery 10, no. 1: Portico. 10.1002/ams2.846. PMC10170370 37179542

[crf370297-bib-0126] Wärnberg, J. , A. Marcos , G. Bueno , and L. A. Moreno . 2009. “Functional Benefits of Psyllium Fiber Supplementation.” Current Topics in Nutraceutical Research 7, no. 2: 55–64.

[crf370297-bib-0127] Yadav, N. , V. Sharma , S. Kapila , R. Kumar Malik , and S. Arora . 2016. “Hypocholesterolaemic and Prebiotic Effect of Partially Hydrolysed Psyllium Husk Supplemented Yoghurt.” Journal of Functional Foods 24: 351–358. 10.1016/j.jff.2016.04.028.

[crf370297-bib-0147] Yang, C. , S. Liu , H. Li , et al. 2021. “The Effects of Psyllium Husk on Gut Microbiota Composition and Function in Chronically Constipated Women of Reproductive Age Using 16S rRNA Gene Sequencing Analysis.” Aging 13, no. 11: 15366–15383. 10.18632/aging.203095. 34081625 PMC8221300

[crf370297-bib-0128] Ye, Z.‐H. , and R. Zhong . 2022. “Outstanding Questions on Xylan Biosynthesis'.” Plant Science 325: 111476. 10.1016/j.plantsci.2022.111476. 36174800

[crf370297-bib-0129] Yin, J.‐Y. , H.‐H. Chen , H.‐X. Lin , M.‐Y. Xie , and S.‐P. Nie . 2016. “Structural Features of Alkaline Extracted Polysaccharide From the Seeds of *Plantago asiatica* L. and Its Rheological Properties.” Molecules 21, no. 9: 1181. 10.3390/molecules21091181. 27608001 PMC6273411

[crf370297-bib-0130] Yu, L. , H. Lutterodt , and Z. Cheng . 2009. “Beneficial Health Properties of Psyllium and Approaches to Improve Its Functionalities.” Advances in Food and Nutrition Research 55: 193–220. 10.1016/S1043-4526(08)00404-X. 18772105

[crf370297-bib-0131] Yu, L. , J. Perret , T. Parker , and K. G. D. Allen . 2003. “Enzymatic Modification to Improve the Water‐Absorbing and Gelling Properties of Psyllium'.” Food Chemistry 82, no. 2: 243–248. 10.1016/S0308-8146(02)00520-4.

[crf370297-bib-0132] Yu, L. , J. R. Stokes , and G. E. Yakubov . 2021. “Viscoelastic Behaviour of Rapid and Slow Self‐Healing Hydrogels Formed by Densely Branched Arabinoxylans From *Plantago ovata* Seed Mucilage.” Carbohydrate Polymers 269: 118318. 10.1016/j.carbpol.2021.118318. 34294330

[crf370297-bib-0133] Yu, L. , G. E. Yakubov , E. P. Gilbert , K. Sewell , A. M. L. V. D. Meene , and J. R. Stokes . 2019. “Multi‐Scale Assembly of Hydrogels Formed by Highly Branched Arabinoxylans From *Plantago ovata* Seed Mucilage Studied by USANS/SANS and Rheology.” Carbohydrate Polymers 207: 333–342. 10.1016/j.carbpol.2018.11.098. 30600014

[crf370297-bib-0134] Yu, L. , G. E. Yakubov , M. Martínez‐Sanz , E. P. Gilbert , and J. R. Stokes . 2018. “Rheological and Structural Properties of Complex Arabinoxylans From *Plantago ovata* Seed Mucilage Under Non‐Gelled Conditions.” Carbohydrate Polymers 193: 179–188. 10.1016/j.carbpol.2018.03.096. 29773370

[crf370297-bib-0135] Yu, L. , G. E. Yakubov , W. Zeng , et al. 2017. “Multi‐Layer Mucilage of *Plantago ovata* Seeds: Rheological Differences Arise From Variations in Arabinoxylan Side Chains.” Carbohydrate Polymers 165: 132–141. 10.1016/j.carbpol.2017.02.038. 28363533

[crf370297-bib-0136] Yu, L. L. , H. Lutterodt , and Z. Cheng . 2008. “Beneficial Health Properties of Psyllium and Approaches to Improve Its Functionalities.” Advances in Food and Nutrition Research 55: 193–220. 10.1016/S1043-4526(08)00404-X 18772105

[crf370297-bib-0137] Zhang, H. , S. Sun , and L. Ai . 2022. “Physical Barrier Effects of Dietary Fibers on Lowering Starch Digestibility.” Current Opinion in Food Science 48: 100940. 10.1016/j.cofs.2022.100940.

[crf370297-bib-0138] Zhang, J. , C. Wen , H. Zhang , and Y. Duan . 2019. “Review of Isolation, Structural Properties, Chain Conformation, and Bioactivities of Psyllium Polysaccharides.” International Journal of Biological Macromolecules 139: 409–420. 10.1016/j.ijbiomac.2019.08.014. 31381918

[crf370297-bib-0139] Zhang, X. , Y. Zhao , Y. Li , L. Zhu , Z. Fang , and Q. Shi . 2020. “Physicochemical, Mechanical and Structural Properties of Composite Edible Films Based on Whey Protein Isolate/Psyllium Seed Gum.” International Journal of Biological Macromolecules 153: 892–901. 10.1016/j.ijbiomac.2020.03.018. 32142843

[crf370297-bib-0140] Zhao, H. , Q. Wang , Y. Sun , et al. 2014. “Purification, Characterization and Immunomodulatory Effects of *Plantago depressa* Polysaccharides.” Carbohydrate Polymers 112: 63–72. 10.1016/j.carbpol.2014.05.069. 25129717

[crf370297-bib-0141] Zhong, R. , D. Cui , D. R. Phillips , N. T. Sims , and Z.‐H. Ye . 2021. “Functional Analysis of GT61 Glycosyltransferases From Grass Species in Xylan Substitutions.” Planta 254, no. 6: 131. 10.1007/s00425-021-03794-y. 34821996

[crf370297-bib-0142] Zhou, P. , M. Eid , W. Xiong , et al. 2020. “Comparative Study Between Cold and Hot Water Extracted Polysaccharides From *Plantago ovata* Seed Husk by Using Rheological Methods'.” Food Hydrocolloids 101: 105465. 10.1016/j.foodhyd.2019.105465.

[crf370297-bib-0143] Zhou, P. , L. Wen , T. Ai , H. Liang , J. Li , and B. Li . 2022. “A Novel Emulsion Gel Solely Stabilized by the Hot Water Extracted Polysaccharide From Psyllium Husk: Self‐Healing Plays a Key Role.” Food Hydrocolloids 130: 107718. 10.1016/j.foodhyd.2022.107718.

[crf370297-bib-0144] Zhou, Z. , F. Ye , L. Lei , S. Zhou , and G. Zhao . 2022. “Fabricating Low Glycaemic Index Foods: Enlightened by the Impacts of Soluble Dietary Fibre on Starch Digestibility.” Trends in Food Science & Technology 122: 110–122. 10.1016/j.tifs.2022.02.016.

[crf370297-bib-0145] Ziemichód, A. , M. Wójcik , and R. Różyło . 2019. “Seeds of *Plantago psyllium* and *Plantago ovata*: Mineral Composition, Grinding, and Use for Gluten‐Free Bread as Substitutes for Hydrocolloids.” Journal of Food Process Engineering 42, no. 1: e12931. 10.1111/jfpe.12931.

